# Supplementary material for: Long-Range Signaling Activation and Local Inhibition Separate the Mesoderm and Endoderm Lineages
Source: Dev Cell. 2018 Jan 22;44(2):179–191.e5. doi: 10.1016/j.devcel.2017.11.021 (PMC5791662; doi:10.1016/j.devcel.2017.11.021)
Supplement: Document S1. Figures S1–S6 [file mmc1.pdf]

Developmental Cell, Volume *44*

Supplemental Information

Long-Range Signaling Activation and Local Inhibition Separate the Mesoderm and Endoderm Lineages

Antonius L. van Boxtel, Andrew D. Economou, Claire Heliot, and Caroline S. Hill

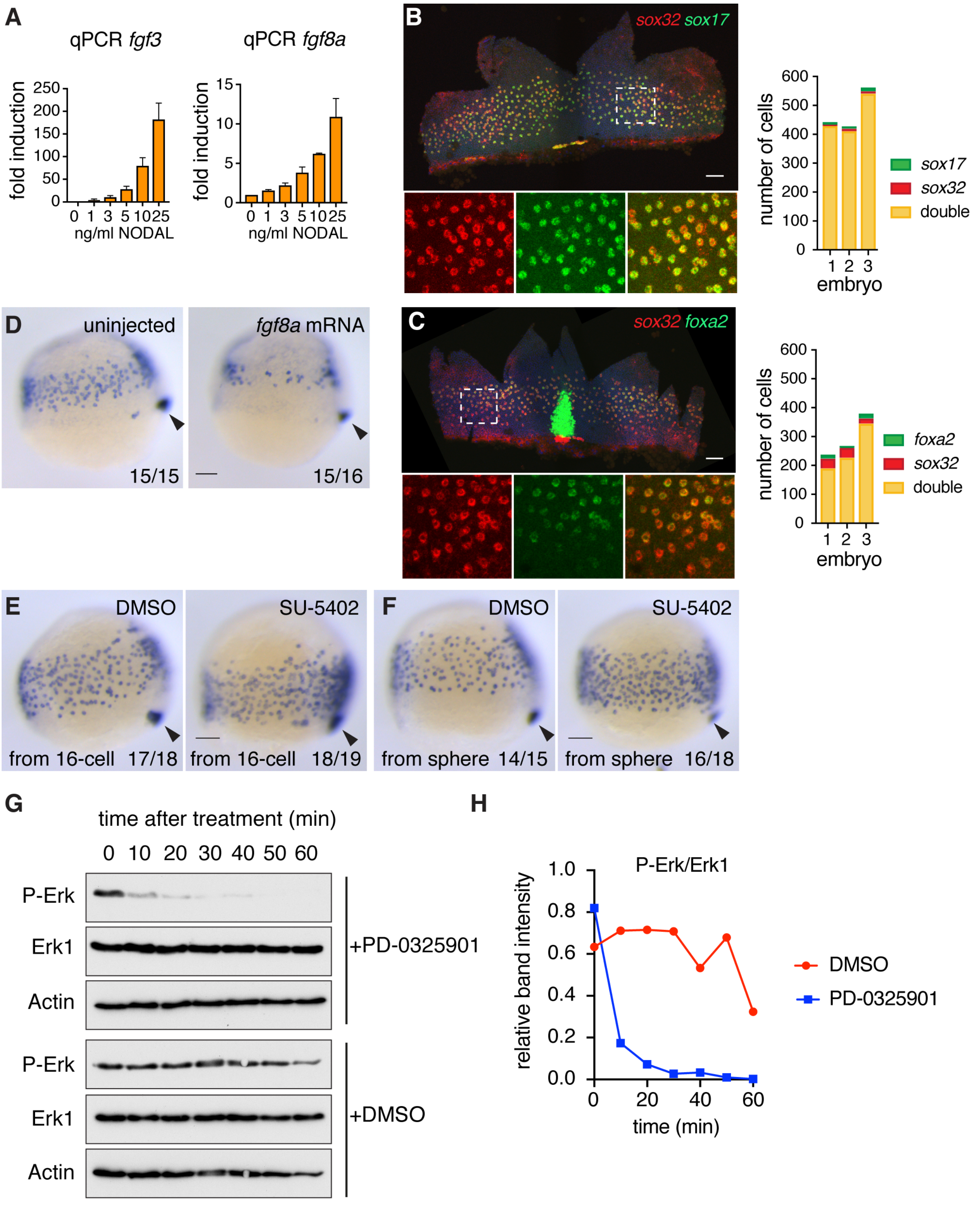

Figure S1, related to Figure 1. Fgf signaling inhibits endoderm specification

(A) qPCR for *fgf3* and *fgf8a* in zebrafish dissociated embryos treated with increasing concentrations of NODAL, mean ± SEM, n >3.

(B) Left panel: Flat-mounted 80% epiboly embryo stained for *sox32* and *sox17*. Box with dashed white line indicates the enlarged area presented in the bottom panels where the staining

is shown for the individual genes and the two combined. Right panel: quantification of endoderm progenitors from three embryos stained as in left panel.

(C) As in (B) but for *sox32* and *foxa2* in 70% epiboly embryos. The strong *foxa2* staining in the center of the flat-mount image corresponds to the *foxa2* expressing axial mesoderm.

(D) Representative images of either uninjected or *fgf8a* mRNA-injected embryos (10 pg) isolated at 75% epiboly and stained for *sox17* by WISH. Overexpression of Fgf8a results in reduced endodermal cell numbers. Arrowhead, dorsal forerunner cells, which mark the most posterior end of the embryo to compare staging.

(E) Representative images of 75% epiboly embryos stained for *sox17* by WISH after treatment with either DMSO as control or 10 μM SU-5402. Chemicals were added from the 16-cell stage. Arrowhead, dorsal forerunner cells. Note that as well as increasing *sox17*-positive endoderm progenitors, SU-5402 also partially inhibits cell migration.

(F) Same as in (E) but for embryos treated from sphere stage. Note that Fgf signaling inhibition in (E) and (F) both result in more endodermal progenitors.

(G) 50% epiboly embryos were treated with DMSO or PD-0325901 for the times shown. Whole cell extracts were Western blotted for P-Erk, Erk1 and Actin as a loading control.

(H) The Western blots in (G) were scanned and quantitated using ImageJ.

All scale bars are 100 μm.

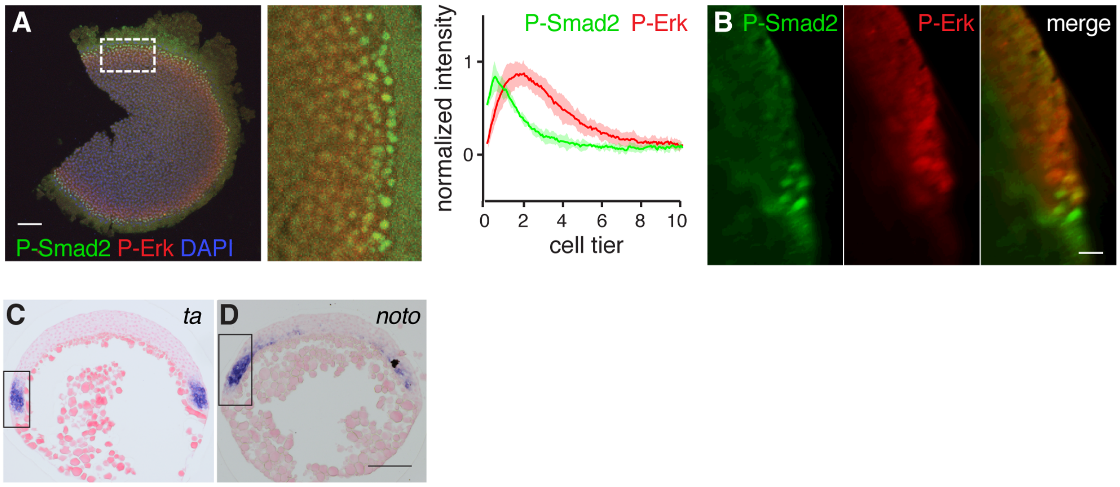

Figure S2, related to Figure 2. Cells directly adjacent to the YSL maintain low levels of P-Erk

(A) Example of flat-mounted, 50% epiboly embryo, stained by immunohistochemistry for P-Erk and P-Smad2. Right panel shows average of intensity traces of three embryos (mean ± SD). Scale bar, 100 µm.

(B) Resliced optical section of 50% epiboly embryo stained as in (A) obtained with light sheet microscope. Note that nuclei close to the YSL have high P-Smad2, but low P-Erk levels. Scale bar, 25 µm.

(C) Section of paraffin embedded WISH-stained 50% embryo for *ta*. Dorsal to the right. Box indicates enlarged area in Figure 2G. Scale bar, 100 µm.

(D) As in (C) for *noto,* except that dorsal is to the left.

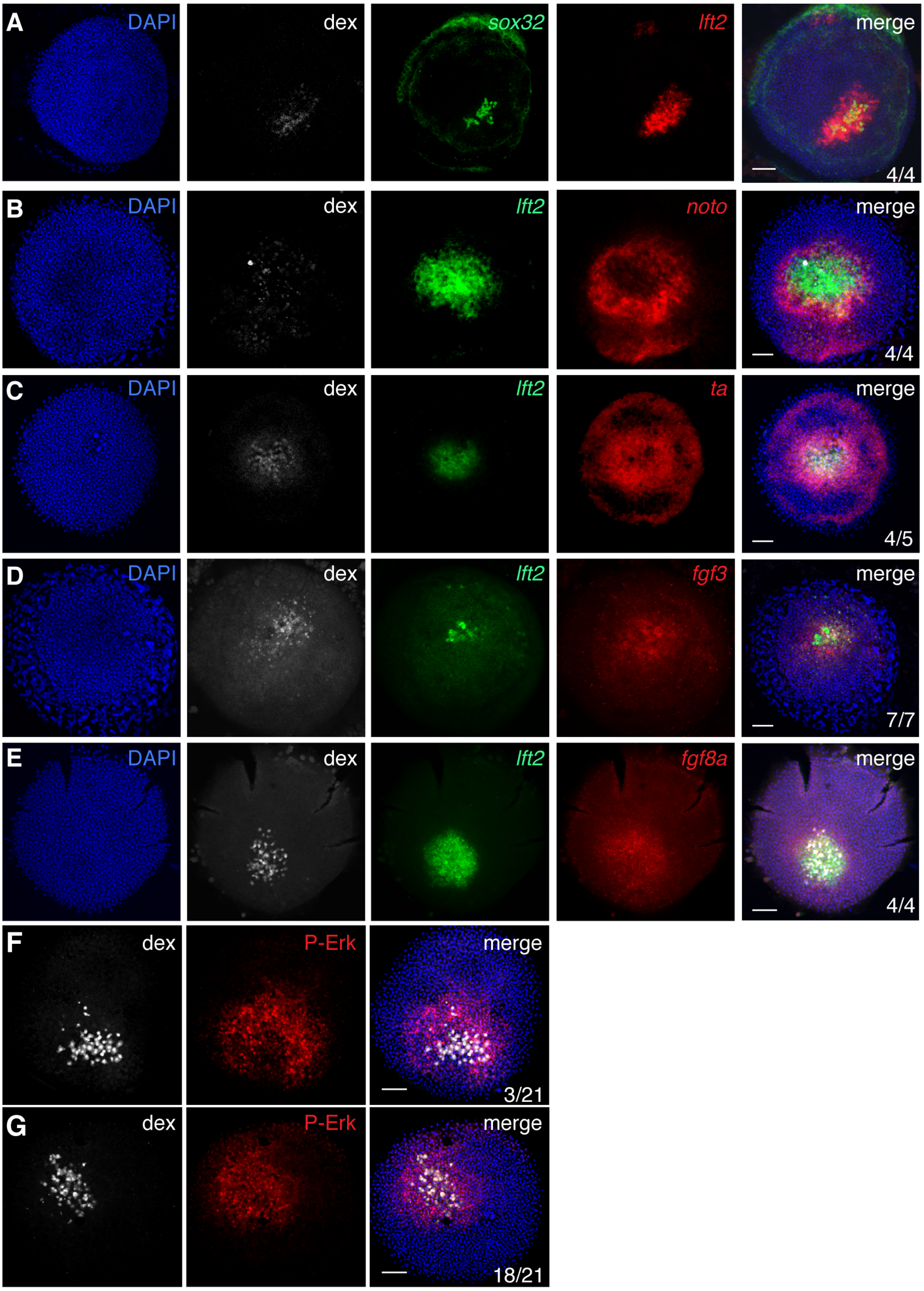

Figure S3, related to Figure 3. Nodal-induced patterning in the animal pole mimics patterning in the margin

(A) Flat-mounted germ ring stage embryos containing a Nodal-expressing clone stained for *sox32* and *lft2*. This is the same embryo shown in Figure 3B left panel, but here the individual

channels are shown. The Nodal-expressing clones are visualized with the dextran fluorescein (dex), and nuclei are visualized with DAPI. A merge of all channels is also shown. Note that *sox32*-positive cells are specified within the *lft2*-positive domain. In the bottom right corner, the number of representative embryos is depicted out of total analyzed embryos from two independent experiments.

(B) As in (A) but for *lft2* and *noto*. *noto* expression is lower in the *lft2* expressing domain. This is the same embryo shown in Figure 3B, middle panel.

(C) As in (A) but for *lft2* and *ta.* Expression of *ta* is lower within the *lft2* expressing domain. This is the same embryo shown in Figure 3B, right panel.

(D) As in (A) but for *lft2* and *fgf3.* This is the same embryo shown in Figure 3C, left panel.

(E) As in (A) but for *lft2* and *fgf8a.* This is the same embryo shown in Figure 3C, right panel.

(F and G) Examples of Nodal-expressing clones visualized with dextran fluorescein (dex) and stained for P-Erk. In very dense clones (3/21) it is clear that the cells expressing Nodal show a clear domain of low P-Erk staining (F). In other clones (18/21), the P-Erk staining is heterogeneous throughout (G).

All scale bars are 100 µm.

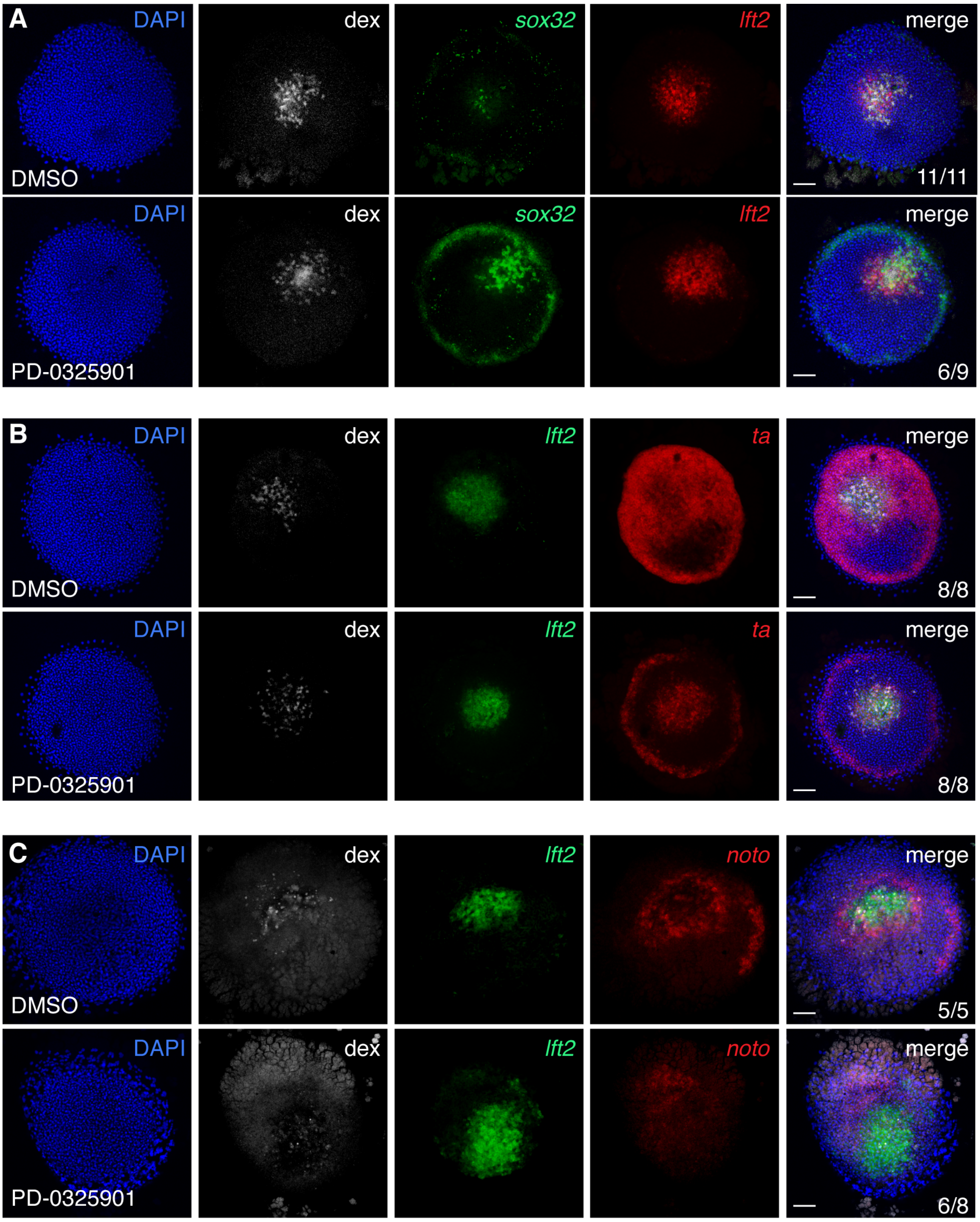

Figure S4, related to Figure 4. Inhibition of P-Erk signaling promotes the formation of *sox32*-positive cells in Nodal-expressing clones

(A) Flat-mounted germ ring stage embryos containing a Nodal-expressing clone treated with DMSO or MEK inhibitor PD-0325901 and stained for *sox32* and *lft2*. These are the same embryos as in Figure 4A, except that here individual channels are shown. The number of *sox32*-positive cells is strongly increased in the PD-0325901-treated embryo.

(B) As in (A), but for *lft2* and *ta*. These are the same embryos as in Figure 4D.

(C) As in (A), but for *lft2* and *noto*. These are the same embryos as in Figure 4E. In (B) and (C), incubation with PD-0325901 inhibits expression of *ta* and *noto* around the clone, confirming that this expression is due to Fgf signaling.

All scale bars are 100 μm.

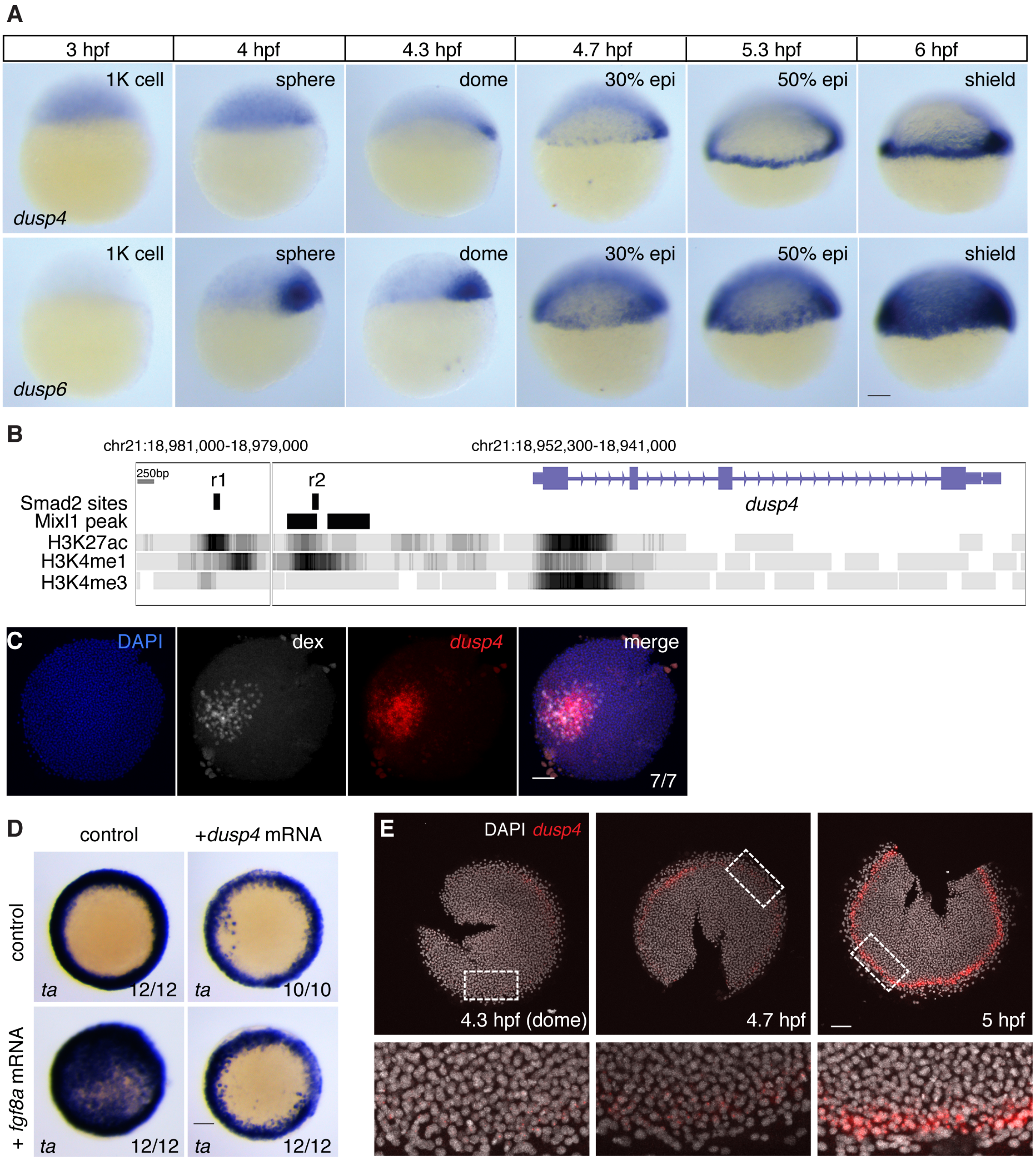

Figure S5, related to Figure 5. Zebrafish *dusp4* is expressed in the first two cell tiers and dephosphorylates Erk

(A) WISH for *dusp4* and *dusp6* expression in 1000-cell (1K) to shield stage embryos. Both *dusp4* and *dusp6* are initially transiently expressed in a dorsal domain, but from 30% epiboly start to be expressed in the ventral and lateral margin. *dusp4* expression remains confined up to the first cell tiers whereas *dusp6* is expressed in a much larger domain. The hours post fertilization (hpf) are indicated above.

(B) The *dusp4* locus from the UCSC genome browser (Zv9) showing the H3K27ac, H3K4me1 and H3K4me3 tracks. The positions of the putative enhancers r1 and r2 are shown, as is the position of the Mixl1 peaks taken from data reported in Nelson et al., 2017.

(C) FISH for *dusp4* mRNA surrounding Nodal-expressing clone marked with dextran fluorescein (dex). This is the same embryo shown in Figure 5G.

(D) WISH for *ta* after *fgf8a* and/or *dusp4* mRNA injection. Embryos are from the same experiment as the Western blot in Figure 5H.

(E) FISH for *dusp4* in flat-mounted embryos fixed at the times indicated to show how expression is induced over time from dome stage (4.3 hpf). Note that staining is seen in 2–3 cell tiers from the YSL at the 5 hpf. These are representative embryos from those used to calculate the traces in Figure 5I.

All scale bars are 100 µm.

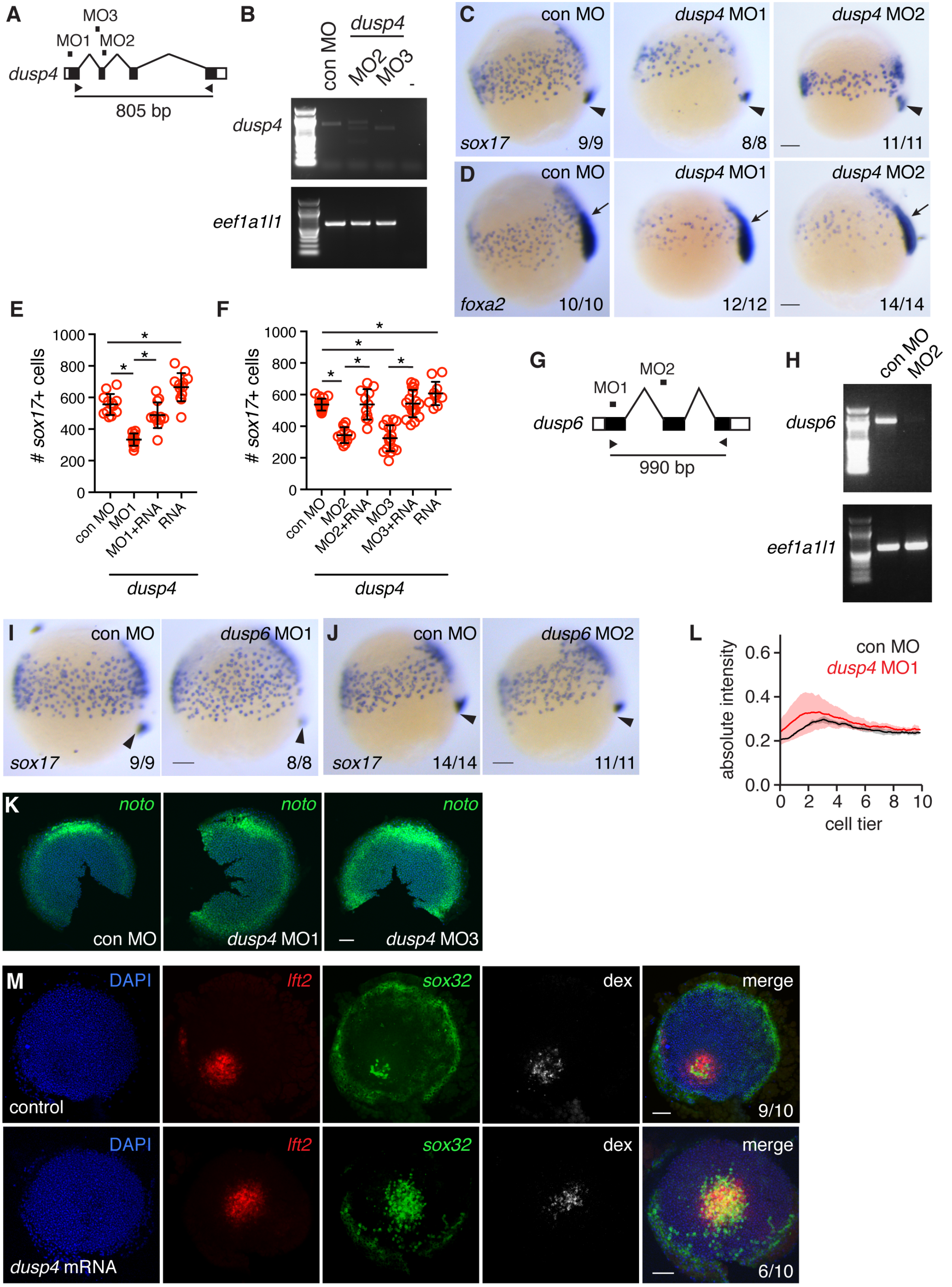

Figure S6, related to Figure 6. *dusp4,* but not *dusp6* loss-of-function, reduces endoderm specification

(A) Schematic representation of knockdown strategy for *dusp4* using three non-overlapping MOs (MO1–3).

(B) For MO2 and MO3, RT-PCR was performed using the indicated oligonucleotides shown in (A) to generate an 805 bp band. The housekeeping gene *eef1a1l1* was used as a control. Note that both splice blocking MOs result in loss of the 805 bp band.

(C) WISH for *sox17* on 75% epiboly embryos after injection with MO1 and MO2. Arrowhead, dorsal forerunner cells.

(D) As in (C) for *foxa2*. Note reduction of both *foxa2* and *sox17* with *dusp4* knockdown, reflecting reduction of the entire endodermal cell population (see also Figure S1B,C). Arrow, *foxa2* expression in the axial mesoderm.

(E) Quantification of endodermal cell numbers at 75% epiboly in embryos injected with control MO (con MO), *dusp4* MO1 ± 400 pg *dusp4* mRNA containing 5 silent mutations in the MO1 binding site, or with the mutated *dusp4* mRNA alone. (Means ± SD, * Mann-Whitney U test, $p < 0.05$) The MO1 phenotype is partially rescued with the mRNA.

(F) Quantification of endodermal cell numbers at 75% epiboly in embryos injected with control MO (con MO), *dusp4* MO2 or MO3 ± 400 pg *dusp4* mRNA, or with the *dusp4* mRNA alone. (Means ± SD, * Mann-Whitney U test, $p < 0.05$) The morphant phenotype is partially rescued with the mRNA.

(G) Schematic representation of knockdown strategy for *dusp6* using two non-overlapping MOs (MO1 and MO2).

(H) RT-PCR for *dusp6* after knock down with MO2.

(I) WISH for *sox17* with *dusp6* MO1 at 75% epiboly.

(J) As in (I) but using *dusp6* MO2. Neither of the *dusp6* MOs showed any effect on endodermal cell numbers. In (I) and (J), arrowhead marks dorsal forerunner cells.

(K) Flat-mounted 50% epiboly embryos stained for *noto* after knockdown of *dusp4*.

(L) Quantification of *noto* profile after injection with *dusp4* MO1 compared to control MO. For each condition, n=3, means are shown by the lines and the shading indicates the SD.

(M) Flat-mounted germ ring stage embryos containing a Nodal clone that were either control injected or injected with *dusp4* mRNA and stained for *sox32* and *lft2.* These are the same embryos as shown in Figure 6F, but here individual channels are shown.

All scale bars are 100 µm.
