# Supplementary material for: Long-Range Signaling Activation and Local Inhibition Separate the Mesoderm and Endoderm Lineages
Source: Dev Cell. 2018 Jan 22;44(2):179–191.e5. doi: 10.1016/j.devcel.2017.11.021 (PMC5791662; doi:10.1016/j.devcel.2017.11.021)
Supplement: Document S2. Article plus Supplemental Information [file mmc3.pdf]

Developmental Cell

Article

# Long-Range Signaling Activation and Local Inhibition Separate the Mesoderm and Endoderm Lineages

## Graphical Abstract

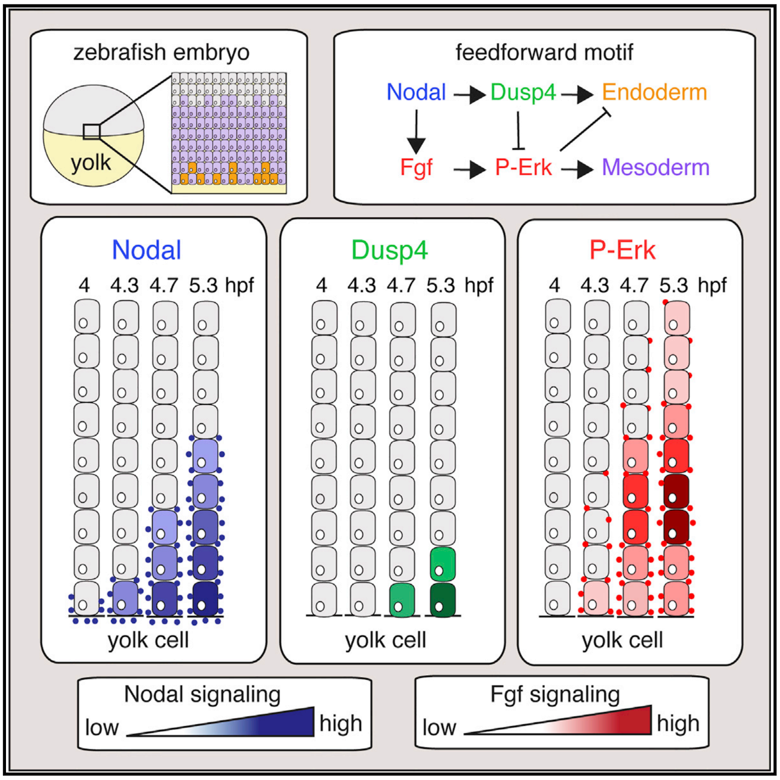


## Highlights

- Nodal-induced Fgf signaling via P-Erk inhibits endoderm specification
- P-Erk levels are attenuated where endodermal progenitors are specified
- Nodal-induced Dusp4 locally attenuates P-Erk to allow endoderm specification
- This incoherent feedforward motif underlies separation of endoderm from mesoderm

## Authors

Antonius L. van Boxtel, Andrew D. Economou, Claire Heliot, Caroline S. Hill

## Correspondence

caroline.hill@crick.ac.uk

## In Brief

van Boxtel et al. uncover a feedforward patterning event that underlies the separation of mesoderm and endoderm lineages in zebrafish. In this system, Nodal signaling in marginal cells induces long-range Fgf signaling, which promotes mesodermal fate and inhibits endoderm induction. Simultaneously, Nodal-induced local Fgf inhibition through Dusp4 allows endoderm specification.

van Boxtel et al., 2018, Developmental Cell *44*, 179–191
January 22, 2018 © 2017 The Authors. Published by Elsevier Inc.
https://doi.org/10.1016/j.devcel.2017.11.021

Developmental Cell
Article

# Long-Range Signaling Activation and Local Inhibition Separate the Mesoderm and Endoderm Lineages

Antonius L. van Boxtel,[1,2] Andrew D. Economou,[1,2] Claire Heliot,[1] and Caroline S. Hill[1,3,*]
[1]Developmental Signalling Laboratory, The Francis Crick Institute, 1 Midland Road, London NW1 1AT, UK
[2]These authors contributed equally
[3]Lead Contact
*Correspondence: caroline.hill@crick.ac.uk
https://doi.org/10.1016/j.devcel.2017.11.021

## SUMMARY

**Specification of the three germ layers by graded Nodal signaling has long been seen as a paradigm for patterning through a single morphogen gradient. However, by exploiting the unique properties of the zebrafish embryo to capture the dynamics of signaling and cell fate allocation, we now demonstrate that Nodal functions in an incoherent feedforward loop, together with Fgf, to determine the pattern of endoderm and mesoderm specification. We show that Nodal induces long-range Fgf signaling while simultaneously inducing the cell-autonomous Fgf signaling inhibitor Dusp4 within the first two cell tiers from the margin. The consequent attenuation of Fgf signaling in these cells allows specification of endoderm progenitors, while the cells further from the margin, which receive Nodal and/or Fgf signaling, are specified as mesoderm. This elegant model demonstrates the necessity of feedforward and feedback interactions between multiple signaling pathways for providing cells with temporal and positional information.**

## INTRODUCTION

One of the first and most important steps in vertebrate development is the establishment of the three germ layers, ectoderm, mesoderm, and endoderm. In all vertebrates, the transforming growth factor β (TGF-β) superfamily member Nodal is essential for this process and is thought to act in a morphogen gradient. High prolonged Nodal signaling is required for the specification of endoderm, while lower levels are thought to promote mesoderm induction (Hagos and Dougan, 2007; Zorn and Wells, 2009). However, additional signaling pathways, for example Wnt, BMP, and Fgf, operate upstream and downstream of Nodal, and it is poorly understood how these pathways interact with Nodal to allocate cells to the endodermal versus the mesodermal lineages (Kiecker et al., 2016; Schier and Talbot, 2005). This question is of fundamental importance for understanding vertebrate development, but also for future application of directed differentiation of stem cells in regenerative medicine (Tabar and Studer, 2014).

Nodal ligands signal through a serine/threonine kinase receptor complex comprising two copies each of a type I receptor (Acvr1ba, also called Taram-a) and a type II receptor (Acvr2a/b), together with the co-receptor Tdgf1 (also called Oep) (Schier, 2009). Binding of Nodal to its receptors leads to phosphorylation of the intracellular signal transducer Smad2, which subsequently binds Smad4. Smad2-Smad4 complexes then accumulate in the nucleus where, together with additional transcription factors, such as FoxH1 and Mixer, they induce a mesoderm- and endoderm-specific transcriptional program (Wu and Hill, 2009).

In zebrafish, two Nodal ligands, Nodal-related 1 and 2 (Ndr1/2) specify mesoderm and endoderm at the blastula margin between sphere and shield stages (4–6 hr post fertilization [hpf]) (Feldman et al., 1998; Hagos and Dougan, 2007; Sampath et al., 1998). Nodal signaling in the ventral and lateral margin is initiated by Ndr1/2 secreted by the yolk syncytial layer (YSL), which signals to the overlying blastoderm. Since *ndr1/2* are transcriptional targets of the Nodal pathway, signaling spreads away from the YSL to form a graded signaling domain within the first five cell tiers (Dubrulle et al., 2015; van Boxtel et al., 2015). This occurs rapidly in about 1.3 hr between sphere stage (4 hpf) and 50% epiboly (5.3 hpf), and results in a spatial and temporal gradient of Nodal signaling, with cells closest to the YSL signaling for the longest duration. The size of the Nodal signaling domain is determined by the interplay between Ndr1/2, the Nodal antagonists Lefty1 and Lefty2 (Lft1 and Lft2, respectively) and the *miR-430* family of microRNAs (van Boxtel et al., 2015). The consequence of this temporal gradient is that cells directly adjacent to the YSL accumulate the highest levels of phosphorylated Smad2 (P-Smad2).

Interestingly, a Nodal signaling gradient is sufficient to organize a complete embryonic axis at the animal pole, when an opposing BMP gradient is introduced (Xu et al., 2014). This illustrates that Nodal triggers a cascade of signaling pathways that orchestrates morphogenetic events. One of the first pathways activated within this cascade is Fgf signaling, since several Fgf ligands, including *fgf3* and *fgf8a*, are transcriptional targets of the Nodal pathway (Mathieu et al., 2004). Fgf ligands are secreted glycoproteins that bind specific tyrosine kinase receptors (FgfR1a/1b/2) to activate multiple signaling branches, including the Stat1/3/5, Plcγ-Pkc, PI3K-Pkb, and the Ras-Raf-Mek1/2-Erk1/2 pathways (Dorey and Amaya, 2010; Ornitz and Itoh, 2015). Importantly, Fgf signaling is rapidly activated and


© 2017 The Authors. Published by Elsevier Inc.
This is an open access article under the CC BY license (http://creativecommons.org/licenses/by/4.0/).

highly dynamic as it is subject to negative feedback at multiple levels of the pathway. Negative regulators include enzymes such as the dual specificity phosphatase Dusp6 (also called Mkp3), which can dephosphorylate Erk1/2, and the E3 ubiquitin ligase CBL, which functions at the level of the receptor, as well as non-enzymatic antagonists such as Sef and Spry2/4, which inhibit signaling at multiple points in the pathway (Eswarakumar et al., 2005; Korsensky and Ron, 2016; Thisse and Thisse, 2005). The importance of the signaling dynamics of the Fgf pathway in mesoderm and endoderm specification is largely unknown.

Within the zebrafish marginal domain, mesoderm is specified in up to ten cell tiers from the YSL, whereas endoderm is specified predominantly in the first two cell tiers (Ober et al., 2003; Schier and Talbot, 2005). Interestingly, only a subset of cells in the first two cell tiers give rise to endoderm, which results in endodermal progenitors being intermingled with mesodermal progenitors prior to gastrulation (Dickmeis et al., 2001; Kikuchi et al., 2001; Warga and Nusslein-Volhard, 1999). Although it is clear that Nodal signaling is required for both endoderm and mesoderm specification, it is currently not known what molecular mechanisms control the separation of these lineages. In prospective endoderm progenitors, Nodal induces the expression of the endoderm-specific transcription factor and master regulator, Sox32, which, in conjunction with the evolutionary-conserved transcription factors Foxa2, Gata5, and Sox17, initiate an endoderm-specific transcriptional program (Alexander and Stainier, 1999; Dickmeis et al., 2001; Kikuchi et al., 2001; Ober et al., 2003). Mesoderm is marked and specified by transcription factors including the T-box transcription factors Ta (also called No-tail or Brachyury), Eomesa, Tbx16, and the homeobox transcription factor Noto (also called Floating head) (Schulte-Merker et al., 1994; Talbot et al., 1995; Wardle and Papaioannou, 2008). We recently demonstrated that transcription of mesodermal markers beyond the Nodal signaling domain is induced by Fgf signaling, which is activated downstream of Nodal (van Boxtel et al., 2015). However, the activation of Fgf signaling by Nodal presents a paradox with respect to the formation of endoderm, since Fgf signaling is known to inhibit this process (Mizoguchi et al., 2006; Poulain et al., 2006). Given that Nodal induces transcription of Fgf ligands in the first two cell tiers, these cells must be exposed to high levels of Fgf (van Boxtel et al., 2015). Thus, how endoderm is specified in a domain with active Nodal and Fgf signaling remains unsolved.

In this paper we solve this paradox and propose a model for endoderm and mesoderm specification. We demonstrate that it is the Ras/Erk pathway downstream of the Fgf receptors that is responsible for inhibiting endoderm specification. We go on to show that Nodal induces long-range Fgf signaling in the zebrafish margin, while simultaneously inducing an inhibitor of phosphorylated Erk1/2 in the first two cell tiers from the YSL. This incoherent feedforward motif explains the separation of the mesoderm and endoderm lineages.

## RESULTS

### Erk1/2 Signaling Downstream of Fgf Inhibits Endoderm Specification

To understand how Nodal and Fgf signaling interact during endoderm specification, we first determined the relative spatial organization of endodermal progenitors and *fgf* ligand expression within the lateral margin using whole-mount *in situ* hybridization (WISH) and sectioning. At 50% epiboly (5.3 hpf), *sox32*-positive endodermal progenitors are detected predominantly in the first two cell tiers, intermingled with *sox32*-negative cells (Figures 1A and 1B). At this stage, *fgf3* and *fgf8a* are expressed in four to five cell tiers from the YSL, overlapping the domain where the *sox32*-positive cells are found (Figure 1C). Besides expression in the blastoderm, *fgf3* expression is also found in the YSL. Both *fgf3* and *fgf8a* could be readily induced by recombinant human NODAL in dissociated embryonic cells, supporting the view that Fgf ligand expression in the margin is Nodal dependent (Bennett et al., 2007; Mathieu et al., 2004) (Figure S1A). Thus, endodermal progenitors are specified in a domain that expresses *fgf* ligands, which are induced by Nodal.

We next addressed the timing of the inhibitory effects of Fgf on endoderm formation in a quantitative manner. By 75% epiboly (8 hpf), a single population of involuted endodermal progenitors express *sox32*, *foxa2*, and *sox17*, and migrate away from the margin (Figures S1B and S1C). We have subsequently used *sox17* WISH at 75% epiboly to quantitate endodermal progenitor numbers following manipulation of Fgf signaling. Overexpression of *fgf8a* mRNA reduced the number of endoderm progenitors, whereas inhibition of Fgf signaling from the 16-cell stage, using the Fgf receptor inhibitor SU-5402, increased their number (Figures S1D and S1E) (Mizoguchi et al., 2006; Mohammadi et al., 1997; Poulain et al., 2006). Prior to their expression in the margin, the Fgf ligands are expressed dorsally (Furthauer et al., 2004). Since inhibition of dorsal Fgf signaling before sphere stage could potentially disrupt dorsal-ventral patterning, leading to secondary effects on endoderm specification (Poulain et al., 2006; Schier and Talbot, 2005), we tested the effects of inhibiting Fgf signaling from sphere stage onward. This also resulted in an increase in endodermal progenitors, indicating that Fgf signaling inhibits endoderm formation after sphere stage and thus acts directly on cells of the ventral/lateral margin (Figure S1F).

Given that Erk1/2-mediated Fgf signaling is activated downstream of Nodal in the margin, we asked whether the inhibitory effect of Fgf on endoderm formation was dependent on Erk1/2 (Poulain et al., 2006; van Boxtel et al., 2015) using the Mek1/2 inhibitor PD-0325901 to block the Ras-Erk1/2 signaling pathway (Anastasaki et al., 2012). This inhibitor is fast acting and reduces P-Erk to almost negligable levels within 20 min of addition (Figures S1G and S1H). Incubation with PD-0325901 from sphere stage increased endodermal progenitor numbers at 75% epiboly by approximately 40%, confirming that the effects were Erk1/2 dependent (Figures 1D and 1E). We then investigated the timing of the inhibitory effect using PD-0325901, and showed that the number of endoderm progenitors could only be increased if Mek1/2 was inhibited before 50% epiboly (Figure 1F). This narrowed the time window in which Fgf signaling inhibits endoderm formation to between sphere and 50% epiboly, and suggested that the effect was at the level of transcriptional activation of the endodermal master regulators. Indeed, in dissociated embryonic cells, recombinant basic FGF (bFGF) inhibited NODAL-induced expression of *sox32*, *foxa2*, and *gata5* (Figure 1G). As a positive control we used the Nodal target gene *lft2*, which we previously showed was inhibited by Fgf signaling (van Boxtel et al., 2015). In

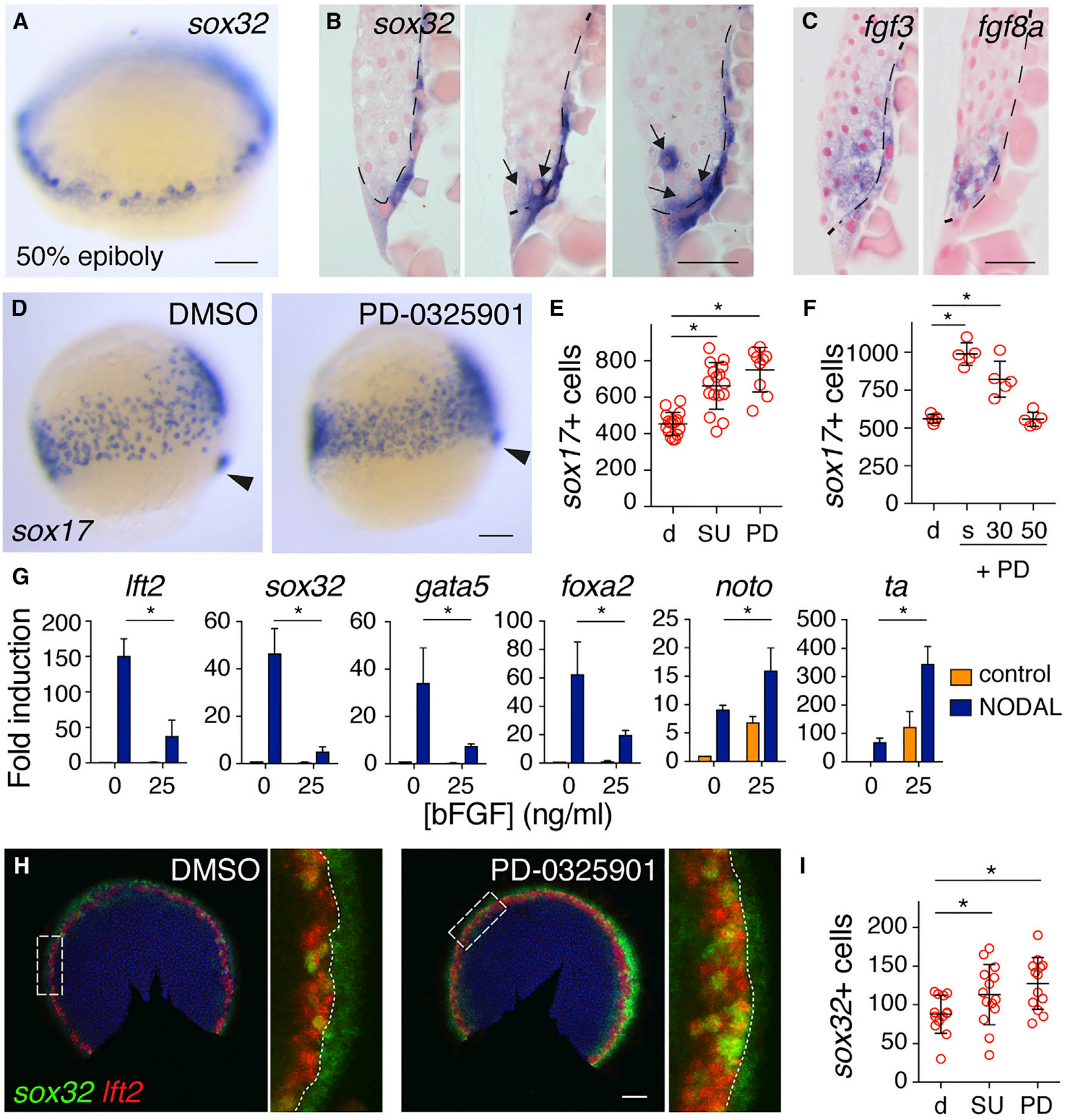


**Figure 1. Endoderm Progenitors Arise in an Fgf Ligand-Expressing Domain, but Their Specification Is Inhibited by Fgf Signaling**

(A) Representative image of a 50% epiboly (5.3 hpf) embryo stained for *sox32* by WISH.

(B) Sections of three 50% epiboly embryos stained for *sox32* by WISH. Dashed line represents the border of the YSL and blastoderm, and arrows indicate *sox32*-positive endoderm progenitors. Scale bar, 25 μm.

(C) Sections of 50% epiboly embryos stained for *fgf3* and *fgf8a*. Note that *fgf3* is also expressed in the YSL. Scale bar, 25 μm.

(D) Images of 75% epiboly (8 hpf) zebrafish embryos treated with DMSO or PD-0325901 from sphere stage, stained for *sox17* by WISH. Arrowhead indicates dorsal forerunner cells to aid comparison of stages.

(E) Quantification of *sox17*-positive cells in embryos as in (D) treated with DMSO (d), SU-5402 (SU), or PD-0325901 (PD). Means ± SD, Mann-Whitney U test; *$p < 0.05$.

(F) Quantification of *sox17*-positive cells at 90% epiboly after treatment with DMSO (d) or PD-0325901 (PD) from sphere (s), 30% epiboly (30), or 50% epiboly (50), corresponding to 4, 4.7, and 5.3 hpf, respectively. Means ± SD, Mann-Whitney U test; *$p < 0.05$.

(G) qPCR for indicated genes in dissociated embryonic cells treated with recombinant human NODAL and/or bFGF. Means ±SEM, n = 3, t test; *$p < 0.05$.

(H) Double fluorescence *in situ* hybridization (FISH) for *lft2* and *sox32* in flat-mounted 50% epiboly embryos treated with DMSO or PD-0325901. White box indicates enlargement in right panel and dotted line marks the boundary between the YSL and margin.

(I) Graph of quantification of *sox32*-positive cells in (H). Means ± SD, Mann-Whitney U test; *$p < 0.05$.

Scale bars, 100 μm unless otherwise stated. See also Figure S1.

contrast, expression of the mesodermal transcription factors *ta* and *noto* was induced by bFGF, indicating that they are Fgf target genes (see below).

To confirm that the negative effect of Fgf signaling on endoderm formation was directly at the level of specification, we quantitated endodermal progenitor cell numbers at 50% epiboly

using fluorescence *in situ* hybridization (FISH) for *sox32* and *lft2* (Dickmeis et al., 2001; Kikuchi et al., 2001). We used *lft2* as a control because it is expressed exclusively in the first two cell tiers from the margin (van Boxtel et al., 2015), thus allowing us to distinguish *sox32*-positive endoderm progenitors from the YSL (Figure 1H). Inhibition of Fgf signaling from sphere stage by PD-0325901 or SU-5402 increased numbers of *sox32*-postive cells at 50% epiboly, as well as the number of *lft2*-positive cells (Figures 1H and 1I). Taken together, our data demonstrate that Fgf signaling, through the Ras-Raf-Mek1/2-Erk1/2 pathway, inhibits specification of endoderm progenitors between sphere and 50% epiboly stages. This is likely at the expense of mesoderm, as Fgf inhibition decreases mesoderm progenitors marked by *ta* (van Boxtel et al., 2015).

## Erk1/2-Mediated Fgf Signaling Is Inhibited in the First Two Cell Tiers

Given that *fgf* ligands are induced by Nodal in the first four to five cell tiers from the YSL, and that Erk1/2-mediated Fgf signaling represses endoderm specification, it is remarkable that endoderm is specified in the first two cell tiers at all. To gain insight into this problem, we determined the relative levels of Nodal and Fgf signaling within the margin at 50% epiboly. To this end, we used immunohistochemistry for P-Smad2 and P-Erk, respectively, and quantitated staining intensities within the first ten cell tiers of the margin. As shown previously, nuclear P-Smad2 staining extended up to four to five cell tiers, with highest levels in the first two cell tiers (Figures 2A and 2B) (Dubrulle et al., 2015; van Boxtel et al., 2015). In contrast, nuclear P-Erk staining extended up to around nine to ten cell tiers, but crucially was relatively low in the first two cell tiers compared with the third and fourth cell tiers (Figures 2A and 2C). Imaging of flat-mounted embryos or using light-sheet microscopy resulted in identical spatial signaling patterns, which excluded possible imaging artifacts (Figures S2A and S2B). These experiments demonstrated that levels of Erk1/2-mediated Fgf signaling were relatively low at 50% epiboly in the two cell tiers closest to the YSL.

We also investigated whether Fgf target gene expression followed a similar spatial distribution pattern as P-Erk. For this experiment we focused on *ta* and *noto*. We previously showed that the *ta* expression beyond the Nodal signaling domain is Fgf dependent (van Boxtel et al., 2015), and we confirmed that the lateral and ventral expression of *noto* is also entirely dependent on Fgf signaling, since it is inhibited by the Fgf receptor inhibitor SU-5402 (Figure 2D). Staining of 50% epiboly embryos for *ta* and *noto* using FISH, followed by quantification in the margin of flat-mounted embryos, revealed that the expression levels of both genes were lower in the two cell tiers closest to the YSL, which correlated well with the observed P-Erk pattern (Figures 2E and 2F). In contrast, *lft2* expression levels peaked in the first two cell tiers. Identical patterns were evident in sections of the ventral and lateral margin from *lft2*, *ta*, and *noto* unsaturated WISH-stained embryos at 50% epiboly (Figures 2G, S2C, and S2D). These experiments showed that *sox32*-positive endoderm progenitors are specified within a low P-Erk domain, which extends two cell tiers from the YSL, and is marked by *lft2*. *noto* and *ta* expression is predominantly induced in a high P-Erk domain, from about two to ten cell tiers (Figure 2H). Our data strongly suggest that Fgf signaling is specifically inhibited in the first two cell tiers, thus allowing endoderm specification.

We then focused on the mechanism underlying the low P-Erk levels in the first two cell tiers. Since Erk1/2-mediated Fgf signaling is highly dynamic, we reasoned that rapid negative feedback within the pathway might explain the low levels of P-Erk in the first two cell tiers. We therefore examined how the Fgf signaling domain evolved during the time period in which endoderm is specified, in particular, between sphere (4 hpf) and 40% epiboly (5 hpf). During this period, the positions of cells within the margin do not change extensively (Dubrulle et al., 2015). Quantitative imaging of P-Erk staining in this time period demonstrated that Fgf signaling expanded rapidly to about nine cell tiers (Figure 2I). Crucially, in the first two cell tiers, P-Erk levels also increased over time, but to a lesser extent compared with the third and fourth cell tiers. Over the same period, Nodal signaling, readout by P-Smad2, gradually expanded away from the YSL to about four cell tiers (Figure 2J). From this analysis, we concluded that the first two cell tiers were relatively unresponsive to Fgf ligands compared with cells further away.

## Nodal and Fgf can Induce Endoderm and Mesoderm Progenitors at the Animal Pole

We reasoned that the low responsiveness of the first two cell tiers to Fgf ligands could be due to their position adjacent to the YSL. For instance, the YSL could potentially secrete Fgf antagonists, or there could be differences in the extracellular matrix composition of these cells. However, we ruled out this possibility as *sox32*-positive endodermal cells are readily induced at the animal pole when a Nodal (Ndr1)-expressing clone was introduced at the 128-cell stage and the embryos were allowed to develop to germ ring stage (5.7 hpf) (Figures 3A, 3B, and S3A) (Chen and Schier, 2001; Poulain et al., 2006; Rodaway et al., 1999). This demonstrates that endoderm specification is independent of the position relative to the YSL, but instead is part of a patterning event downstream of Nodal. Strikingly, we found that endodermal cells were specified within a *lft2*-expressing domain, whereas *noto* and *ta* were expressed at higher levels outside the *lft2* domain, mimicking the spatial organization of mesoderm and endoderm progenitors at the margin (Figures 3B and S3A–S3C). This predicted that Fgf signaling was also activated downstream of Nodal surrounding these clones and, indeed, we could demonstrate that transcription of both *fgf3* and *fgf8a* is induced (Figures 3C, S3D, and S3E). Moreover, we observed activation of both Nodal and Fgf signaling surrounding the clones by staining for P-Smad2 and P-Erk, respectively (Figure 3D). We observed that a subset of P-Smad2-positive cells exhibited low levels of nuclear P-Erk staining. Furthermore, in some dense clones, Nodal expression gave rise to a domain of low P-Erk staining in the center, although in most clones this was not the case, likely due to the inherent dispersion of the Nodal-expressing cells between the 128-cell and germ ring stage (Figures S3F and S3G). In conclusion, patterning surrounding Nodal-expressing clones recapitulates the patterning at the margin (Figures 3E and 2H).

We reasoned that, for endoderm to be specified ectopically at the animal pole, the regulatory mechanism that suppressed Fgf

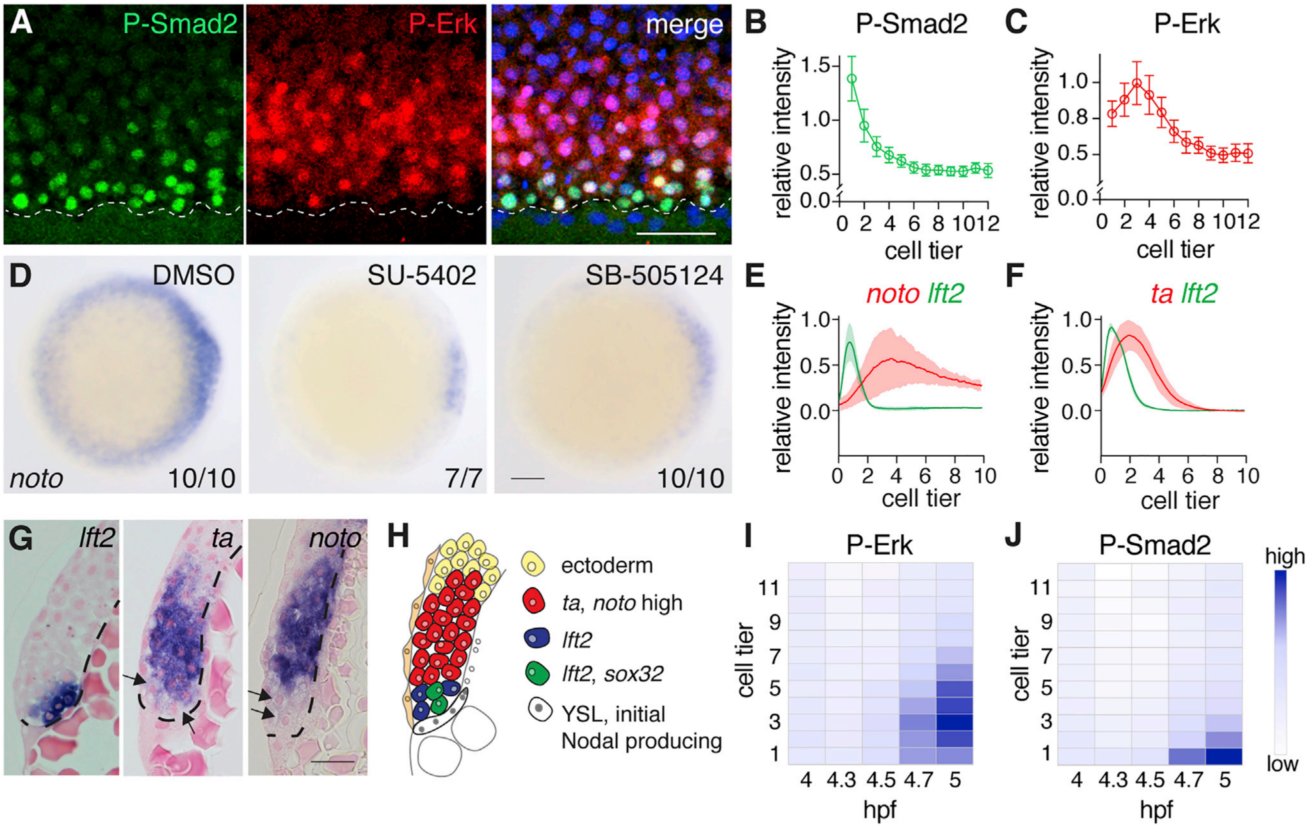


**Figure 2. Cells Adjacent to the YSL Exhibit Low Levels of P-Erk**

(A) Z-Projection of the margin of a 50% epiboly embryo stained for P-Erk and P-Smad2. Dashed line indicates the boundary between the YSL and the margin. Scale bar, 50 μm.

(B) Quantification of three embryos stained for P-Smad2 as in (A). Depicted is the mean ± SD from a representative experiment (n > 3).

(C) As in (B) for P-Erk.

(D) Animal views of WISH-stained 50% epiboly embryos for *noto*, treated with the Fgf signaling inhibitor (SU-5402) or the Nodal signaling inhibitor (SB-505124). Note that the inhibition of *noto* expression by SB-505124 reflects the regulation of Fgf by Nodal. Scale bar, 100 μm.

(E) Quantification of unsaturated FISH-stained embryos for *lft2* and *noto* at 50% epiboly. For each trace n = 3, means are shown by the lines, and the shading indicates the SD.

(F) As in (E), but for *lft2* and *ta*.

(G) Sections of unsaturated WISH-stained embryos for *lft2*, *ta*, and *noto*. Arrows indicate cells with low levels of expression and dashed line marks the border of the YSL. Scale bar, 25 μm.

(H) Schematic representation of gene expression in the margin.

(I) Time course of weighted means of P-Erk staining intensities, normalized to DAPI in the margin from 4 hpf (sphere stage) to 5 hpf (40% epiboly).

(J) As in (I), but for P-Smad2.

See also Figure S2.

signaling also must operate within and surrounding Nodal-expressing clones. We tested this hypothesis by generating Ndr1-expressing clones and incubating the embryos with PD-0325901, followed by quantification of *sox32*-positive endodermal cell numbers at germ ring stage. This clearly demonstrated that inhibition of Fgf signaling led to an increased number of endodermal progenitors around the Ndr1-expressing clones (Figures 4A, 4B, and S4A). Simultaneously, PD-0325901 expanded the size of the *lft2* domain and reduced the expression of the mesodermal markers *ta* and *noto* (Figures 4C–4E, S4B, and S4C). Thus, the signaling events initiated by Ndr1 expression in the animal pole recapitulate the signaling events that underlie patterning of mesoderm and endoderm progenitors at the margin.

## Dusp4 Is a Direct Nodal Target and Dephosphorylates P-Erk

We hypothesized that, for levels of P-Erk to remain low in the first two cell tiers over time, Nodal could simultaneously induce the transcription of *fgf* ligands, as well as an intracellular inhibitor of the Ras-Raf-Mek1/2-Erk1/2 pathway. Interestingly, two dual specificity phosphatases, Dusp4 and Dusp6, which are known to dephosphorylate Erk1/2 in different contexts, are expressed in the margin between the sphere and 50% epiboly stages (Figure S5A) (Bennett et al., 2007; Caunt and Keyse, 2013; Guan and Butch, 1995). We focused on Dusp4 since it is a nuclear phosphatase, whereas Dusp6 is cytoplasmic (Caunt and Keyse, 2013). Dusp4 has also previously been implicated as a regulator of endoderm formation in zebrafish

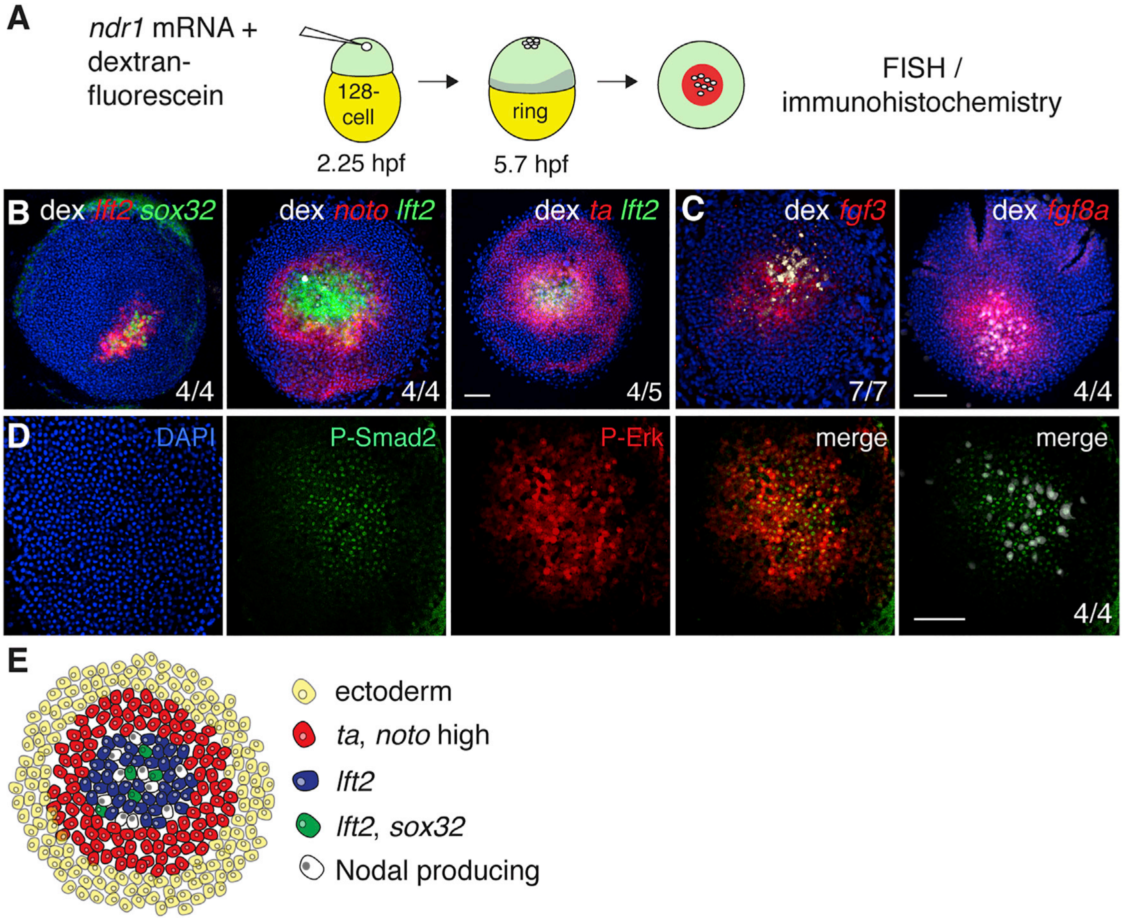


**Figure 3. Ectopic Nodal-Induced Patterning at the Animal Pole Mimics Patterning at the Margin**

(A) Schematic of generation of Ndr1-expressing clones. Single blastomeres are injected at the 128-cell stage (2.25 hpf), fixed at germ ring stage (5.7 hpf) and stained using FISH or immunohistochemistry.

(B) Animal views of germ ring-stage embryos containing Ndr1-expressing clones marked with dextran-fluorescein (dex) and stained for indicated markers.

(C) As in (B), but for *fgf3* and *fgf8a*.

(D) Germ ring-stage embryos containing Ndr1-expressing clones, stained by immunohistochemistry for P-Erk and P-Smad2. Single panels are shown and a merge. The merge on the far right shows the Ndr1-expressing cells labeled with dex (white) with staining for P-Smad2 (green).

(E) Schematic representation of gene expression in surrounding Ndr1-expressing clones.

Scale bars, 100 μm. See also Figure S3.

(Brown et al., 2008). Sectioning of WISH-stained embryos revealed that *dusp4* is expressed in the dorsal YSL and in the first two cell tiers of the ventral and lateral margin at 50% epiboly (Figure 5A). To determine whether *dusp4* is regulated by Nodal or Fgf signaling *in vivo*, we inhibited the pathways with SB-505124 or SU-5402, respectively, and analyzed spatial expression by WISH. For comparison, we also analyzed the expression of *dusp6*, which is known to be regulated by Fgf signaling (Molina et al., 2007). Inhibition of Nodal signaling completely abolished the expression of *dusp4* in the margin, while expression in the YSL was unaffected (Figure 5B). This was not an indirect effect through activation of Fgf signaling, since SU-5402 did not affect the size of the *dusp4* expression domain. In contrast, *dusp6* expression was severely reduced when embryos were treated with SU-5402, but to a lesser extent when treated with SB-505124, likely because of Fgf production by the YSL (Figures 5B and 1C). Moreover, in dissociated embryonic cells, we found that *dusp4*, but not *dusp6,* expression was dose dependently induced by NODAL (Figure 5C). To confirm that *dusp4* is a direct Nodal target gene, we identified two potential enhancer regions (r1 and r2) upstream of the *dusp4* transcriptional start site (TSS) (Figure S5B) and performed chromatin immunoprecipitation for Smad2. Both the r1 and r2 genomic regions, but not the TSS, were bound by Smad2 in a Nodal signaling-dependent manner, similar to a known enhancer site upstream of the Nodal target gene *mixl1* (also called *mixer* or *bon*) (Nelson et al., 2014) (Figure 5D). Furthermore, it was recently reported that Mixl1 was enriched together with Smad2 at site r2 (Nelson et al., 2017) (Figure S5B). Consistent with this, we could demonstrate that expression of *dusp4* was dependent on Mixl1, whereas expression of *lft1*, used as a control, was not (Figures 5E and 5F). Thus, *dusp4* is induced by Nodal via a so-called self-enabling mechanism, where Nodal induces the expression of a transcription factor (Mixl1), which then binds with Smad2 to induce transcription

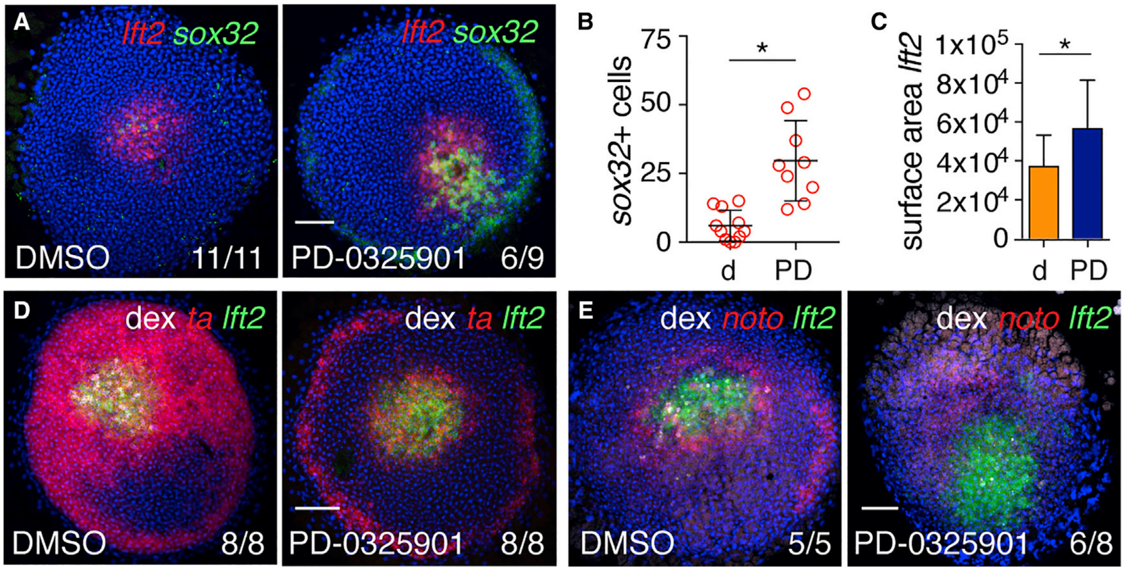


**Figure 4. Fgf Signaling Represses Endoderm Specification around Ndr1 Clones**

(A) Animal views of DMSO- and PD-0325901-treated germ ring-stage embryos containing Ndr1-expressing clones stained for *lft2* and *sox32*.

(B) Quantification of *sox32*-positive cells in (A). Means ± SD, Mann-Whitney U test; *$p < 0.05$.

(C) Quantification of surface area of *lft2*-positive domain surrounding Ndr1-expressing clones in embryos treated as in (A). n = 3, 11 embryos in total. Means ± SD, two-tailed t test; *$p < 0.05$.

(D) Animal views of germ ring-stage embryos treated as in (A), but stained for *ta* and *lft2*.

(E) Animal views of germ ring-stage embryos treated as in (A), but stained for *noto* and *lft2*.

Scale bars, 100 μm. See also Figure S4.

of the target gene (Hill, 2016). *dusp4* was also induced in and around the Nodal-expressing clones in the animal pole (Figures 5G and S5C).

To confirm that zebrafish Dusp4 can dephosphorylate P-Erk *in vivo* we injected *fgf8a* mRNA with or without *dusp4* mRNA and performed western blotting for P-Erk. This demonstrated that Dusp4 attenuates the phosphorylation of Erk (Figure 5H). This was also evident from analysis of *ta* expression using WISH in embryos from the same experiment, where Fgf8a-induced expression of *ta* was attenuated by Dusp4 (Figure S5D). Finally, high-resolution quantitative analysis of *dusp4* expression over time revealed that *dusp4* was first detected at 4.7 hpf, and by 5 hpf (around 40% epiboly) was localized predominantly in the first two cell tiers, co-localizing with *sox32* (Figures 5I, 5J, and S5E). In conclusion, *dusp4* is a direct Nodal target gene that attenuates P-Erk-mediated Fgf signaling and is expressed in the first two cell tiers, correlating with the low P-Erk levels observed in these cells.

## Dusp4 Loss of Function Reduces Endodermal Progenitor Numbers and Increases Fgf Signaling in the Margin

If Dusp4 was responsible for attenuating P-Erk in the first two cell tiers to allow blastomeres to be specified as endoderm, loss of function should result in increased levels of Fgf signaling and reduced endodermal progenitor numbers at 50% epiboly. To determine the effects of Dusp4 depletion, we used a start site morpholino (MO) and two non-overlapping splice-blocking MOs directed against *dusp4* (Figure S6A) (Brown et al., 2008). When injected in one- to two-cell-stage embryos, all three MOs resulted in a reduction of *sox17*- and *foxa2*-positive cells of up to 60% at 75% epiboly (Figures 6A–6C and S6B–S6D). Importantly, co-injection of each *dusp4* MO with capped *dusp4* mRNA partially rescued endodermal progenitor numbers, demonstrating their specificity (Figures S6E and S6F). In contrast, knock down of *dusp6* did not result in a difference in endodermal cell numbers (Figures S6G–S6J). To directly determine the effects of *dusp4* knockdown on attenuation of Fgf signaling, we used WISH and qPCR for *noto* as an integrated Fgf signaling readout. Both experiments showed that the loss of *dusp4* led to higher levels of *noto* expression in the margin at 50% epiboly (Figures 6D, 6E, S6K, and S6L). Finally, since Ndr1-expressing clones induced endoderm specification and *dusp4* was expressed surrounding these clones, the levels of *dusp4* should also control ectopic endodermal cell numbers at the animal pole. Therefore, we generated Ndr1-expressing clones in *dusp4* mRNA-injected embryos and quantitated the number of endodermal cells surrounding these clones at germ ring stage. Indeed, Dusp4 overexpression led to more endoderm progenitors being induced (Figures 6F, 6G, and S6M). From these experiments, we concluded that Dusp4 regulates endoderm specification by attenuating Fgf signaling in the first two cell tiers.

# DISCUSSION

## An Incoherent Feedforward Motif Is Required for the Specification of Endoderm versus Mesoderm

Here we describe a mechanism for the separation of the mesodermal and endodermal lineages in the zebrafish, and show that

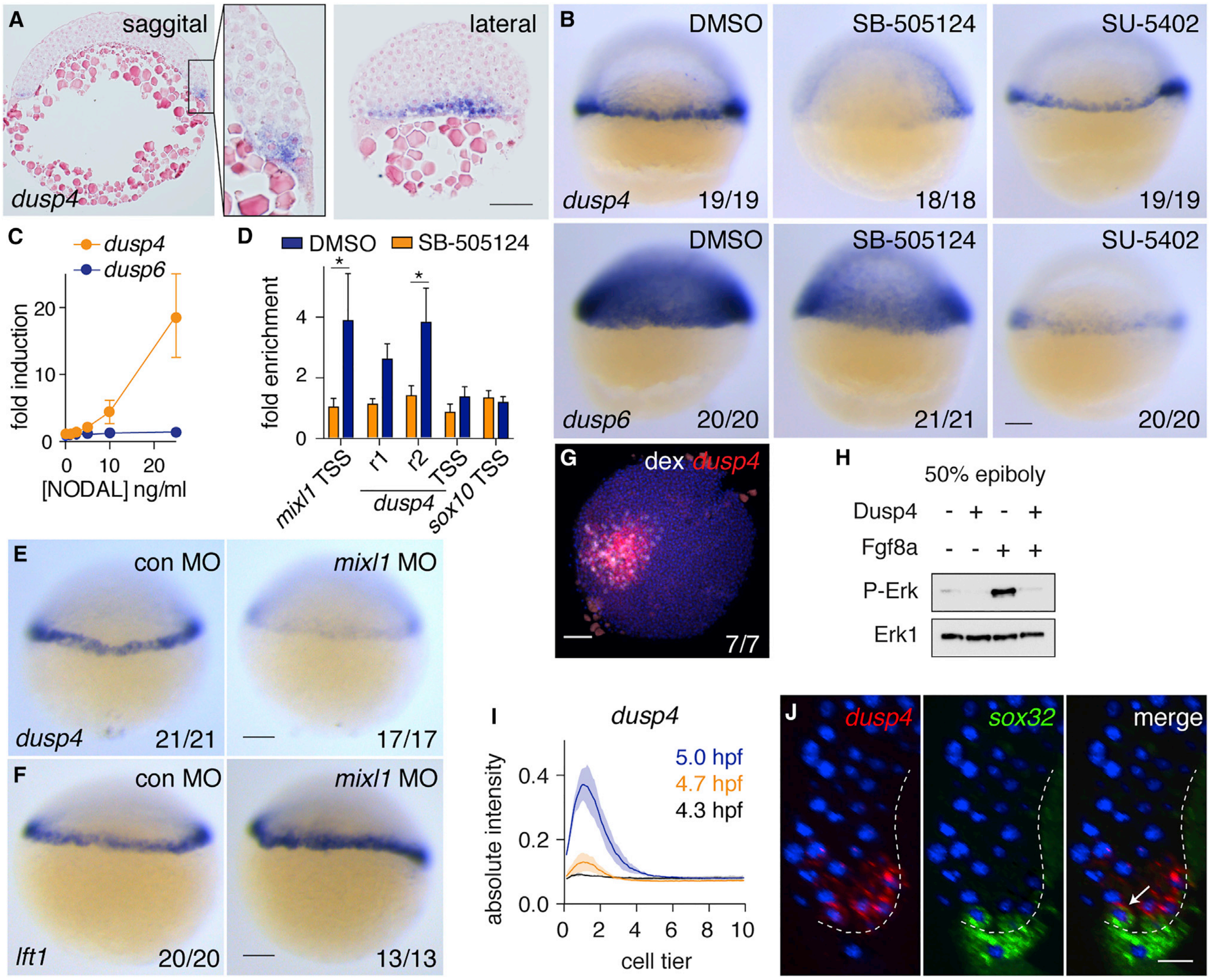


**Figure 5. Zebrafish Dusp4 Is a Direct Nodal Target, Expressed in the First Two Cell Tiers, that Dephosphorylates Erk**

(A) Sagittal and lateral sections of 50% epiboly embryo WISH-stained for *dusp4*.

(B) WISH at 50% epiboly for *dusp4* and *dusp6* after Nodal signaling inhibition (SB-505124) or Fgf signaling inhibition (SU-5402).

(C) qPCR using dissociated embryonic cells treated with increasing NODAL concentrations. Means ± SEM, n = 5.

(D) Chromatin immunoprecipitation for Smad2 on putative enhancers of the *dusp4* gene (r1 and r2) and the *dusp4* transcription start site (TSS) in 50% epiboly embryos treated with DMSO or the Nodal receptor inhibitor SB-505124. The *mixl1* TSS and *sox10* TSS are positive and negative controls respectively. Means ± SEM, n = 4, *$p < 0.05$, t test.

(E and F) 50% epiboly zebrafish embryos injected with control MO (con MO) or *mixl1* MO, stained for *dusp4* (E) or *lft1* (F) by WISH.

(G) Animal view of germ ring-stage embryo containing Ndr1-expressing clone, marked with dextran-fluorescein (dex) and stained for *dusp4.*

(H) Western blot showing attenuation of Fgf8a-induced P-Erk by Dusp4 at 50% epiboly.

(I) Quantification of embryos stained by FISH for *dusp4* at 4.3 (dome), 4.7, and 5.0 hpf. For each time point n = 3, means are shown by the lines and the shading indicates the SD.

(J) A confocal Z-reconstruction of a 50% epiboly embryo stained for *dusp4* and *sox32.* Nuclei are marked by DAPI. Arrow indicates a cell in the blastoderm labeled with both *dusp4* and *sox32*. Dashed white line indicates the border of the YSL. Scale bar, 25 μm.

Scale bars, 100 μm unless otherwise stated. See also Figure S5.

it is not explained by a single morphogen gradient. We provide strong evidence that Nodal triggers a feedforward patterning system, which combines long-range signal activation with local inhibition. Our data demonstrate that Nodal simultaneously induces the expression of secreted Fgf ligands and the cell-autonomous Fgf signaling inhibitor Dusp4 (Figure 7A). This type of wiring pattern has previously been named as an incoherent feedforward motif (Mangan and Alon, 2003), and is widespread in gene regulatory networks, but less known in the context of signaling.

Nodal signaling in the ventral and lateral margin is initiated by the production of Ndr1/2 in the YSL, which induces Nodal signaling in the blastoderm, leading to expression of *ndr1/2* in these cells and subsequent spreading of signaling away from

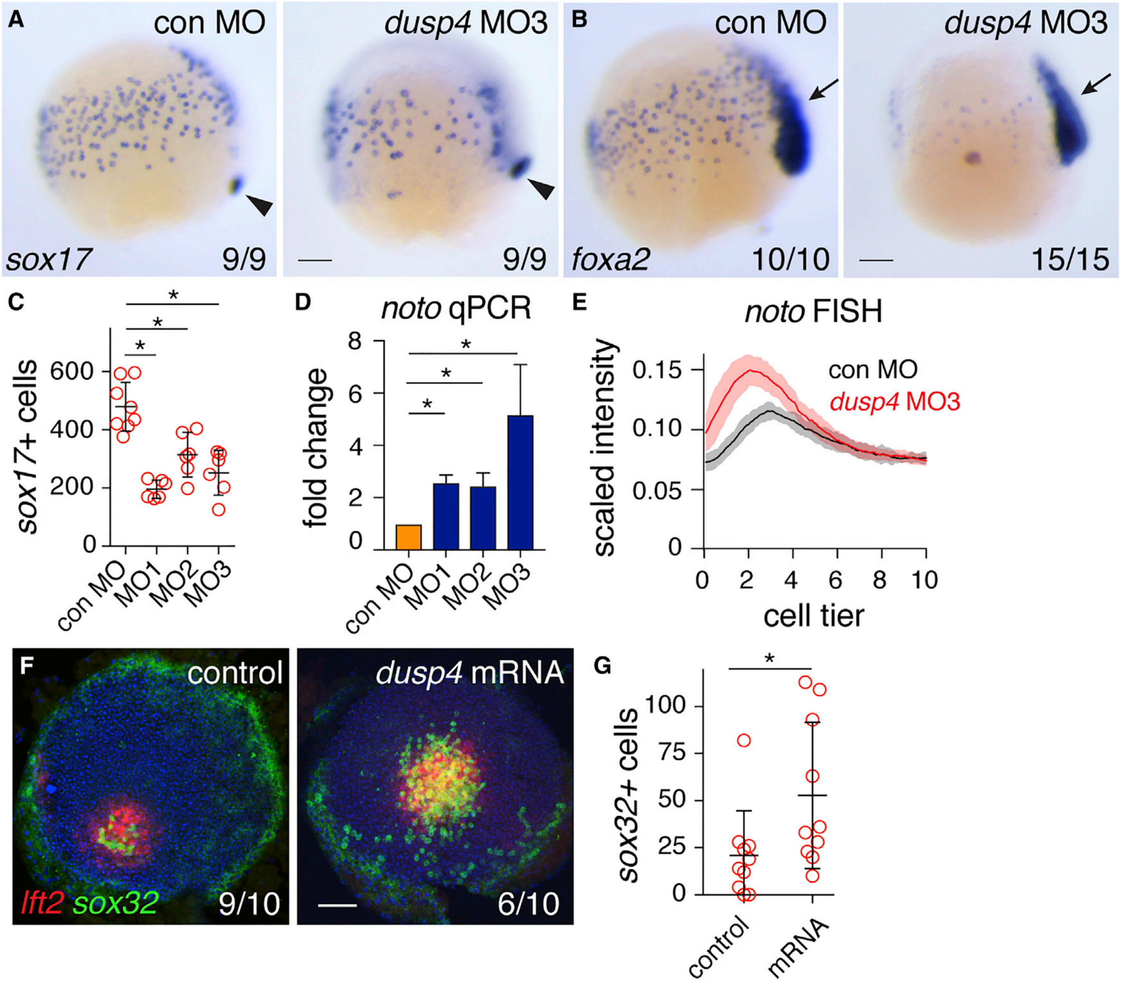


**Figure 6. Dusp4-Mediated Repression of Fgf Signaling Is Required for Endoderm Specification**

(A) Representative images of 75% epiboly zebrafish embryos injected with control MO (con MO) or *dusp4* MO3, stained for *sox17* by WISH. Arrowhead indicates dorsal forerunner cells for comparison of staging.

(B) As in (A), but stained for *foxa2*. Arrow, *foxa2* expression in axial mesoderm.

(C) Quantification of endodermal progenitors in 75% epiboly embryos, injected with indicated *dusp4* MOs. Means ± SD, Mann-Whitney U test; $^*p < 0.05$.

(D) qPCR on 50% epiboly embryos injected with control MO or *dusp4* MOs. Means ± SEM, $n \geq 2$, t test; $^*p < 0.05$.

(E) Traces of *noto* expression detected with FISH in 50% epiboly zebrafish embryos injected with control MO or *dusp4* MO3. For each condition, n = 3. Means are shown by the lines and the shading indicates the SD.

(F) Animal views of germ ring-stage embryos, control-injected or injected with *dusp4* mRNA, containing Ndr1-expressing clone and stained for *lft2* and *sox32*.

(G) Quantification of *sox32*-positive cells as in (F). Means ± SD, Mann-Whitney U test; $^*p < 0.05$.

Scale bars, 100 μm. See also Figure S6.

the YSL. The consequence of this temporal gradient is that cells in the first two cell tiers experience the longest duration of Nodal signaling. As a result, they exhibit the highest levels of P-Smad2, because the P-Smad2 signal is integrated over time (van Boxtel et al., 2015). When Nodal signaling is initiated in the blastoderm, it induces the expression of Fgf ligands, which results in a broad domain of P-Erk activity, up to ten cell tiers. Nodal concurrently induces the expression of the intracellular P-Erk antagonist Dusp4 in the first two cell tiers. Taken together, this results in a pattern where the most marginal cells in tiers 1 and 2 exhibit lower levels of P-Erk compared with cells in tiers 3 and 4, despite being exposed to high levels of Fgf ligands. Cells in the first two cell tiers are therefore protected from the repressive effects of Fgf signaling, experience sustained Nodal signaling, and can thus be specified as endoderm (Figure 7B). Cells further away from the YSL experience higher levels of Erk1/2-mediated Fgf signaling and are specified as mesoderm. We have therefore uncovered a system in which a group of cells are instructed to produce a signaling molecule that they themselves are insensitive to, but which can alter the fate of their neighbors.

### The Importance of the Dynamics of Fgf and Nodal Signaling in the Specification of Endoderm versus Mesoderm

Although Nodal signaling clearly modulates Fgf signaling in the zebrafish margin via the induction of Dusp4, we have found no

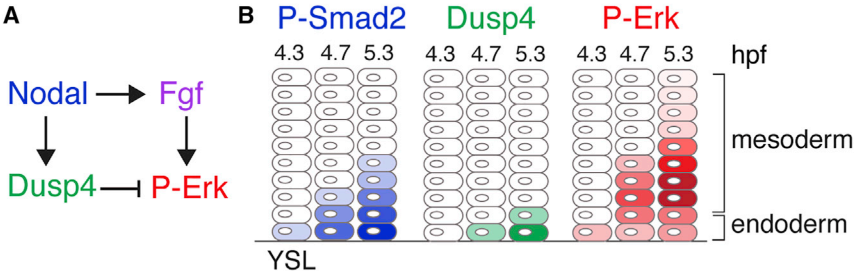


**Figure 7. Model for Meseoderm and Endoderm Lineage Separation through an Incoherent Feedforward Motif**

(A) Wiring diagram for spatial patterning at the margin.

(B) Schematic of growing domains of Nodal and Fgf signaling and *dusp4* expression. Intensity of color reflects signaling levels. Endoderm can be specified in the first cell tiers because of accumulating Nodal signaling, which attenuates Erk-mediated Fgf signaling via Dusp4. Mesoderm is specified by Fgf signaling ± Nodal signaling.

evidence in this system for a direct effect of Fgf signaling on Nodal signaling. Inhibition of Fgf signaling with the receptor inhibitor SU-5402 has no effect on the expression of the Nodal target gene *lft1*, or on the levels of activated P-Smad2 (van Boxtel et al., 2015). Moreover, inhibition of Fgf signaling had no effect on Smad2 linker phosphorylation, which can be induced by Erk1/2 in some contexts (Gao et al., 2009; Kretzschmar et al., 1999) (our unpublished data).

Our data show that attenuation of P-Erk by Nodal-induced Dusp4 is required for endoderm specification, but it is evidently not sufficient as most cells in the first two cell tiers express *dusp4* by 50% epiboly, but not all are specified as endoderm. It is therefore likely that additional mechanisms regulate the stochastic induction of endoderm progenitors at the margin. We have observed that *sox32* expression occurs randomly within the first two cell tiers of the margin, arguing against a lateral inhibition-type mechanism. We speculate that there may be inherent noise in the P-Erk signal, as recent reports have demonstrated stochastic Erk activation pulses in response to growth factors (Aoki et al., 2013). Live imaging of P-Erk will be required to explore this further. Another possibility is that other signaling pathways are involved in generating the salt and pepper pattern of *sox32*-postive cells.

In addition to the role that Fgf signaling plays in stochastic induction of endoderm at the margin, the extended duration of Nodal signaling is also crucial (Hagos and Dougan, 2007; Schier, 2009). The mechanistic reason for endoderm specification requiring extended Nodal signaling may lie in the downstream transcription factor network necessary for *sox32* induction. Transcriptional activation of *sox32* requires a combination of the transcription factors Mixl1, Gata5 (also called Fau), Mezzo, and Eomesa (Bjornson et al., 2005; Du et al., 2012; Kikuchi et al., 2000; Nelson et al., 2017; Poulain and Lepage, 2002; Reiter et al., 2001). Importantly, transcription of *mixl1, gata5*, and *mezzo* requires Nodal signaling (Kikuchi et al., 2000; Poulain and Lepage, 2002; Reiter et al., 2001). This would introduce a short delay in the induction of *sox32* relative to other direct Nodal-induced genes, and thus could explain the requirement for prolonged Nodal signaling.

Another key finding is the restriction of *dusp4* expression to the first two cell tiers at 50% epiboly. Interestingly, *lft2* is also restricted to these cell tiers and we have shown that this is due to inhibition of its transcription by Fgf signaling (van Boxtel et al., 2015). We can rule out this mechanism for *dusp4*, however, as its expression domain does not expand when Fgf signaling is inhibited. One contributing factor in the restricted expression of *dusp4* may be a delay in its transcription. We observe that the domain of *dusp4* expression grows slowly relative to other Nodal targets, being readily detected only at 40% epiboly (5 hpf), compared with genes such as *lft1* and *lft2*, which are expressed in the margin from about dome stage (4.3 hpf) (van Boxtel et al., 2015). Further work will be required to fully elucidate the mechanism.

Our experiments also reveal that the negative effect of Erk1/2-mediated Fgf signaling on endoderm induction is at the level of *sox32* transcription. However, we do not yet know the identity of the targets of Erk phosphorylation that explains this. A possible mechanism could involve one of the transcriptional activators of *sox32* being negatively regulated by Erk phosphorylation. However, Mixl1 and Gata5 are unlikely targets as the loss of *sox32*-positive endodermal progenitors in *bon* and *fau* mutants can be partially rescued by inhibition of Fgf signaling (Mizoguchi et al., 2006). An alternative mechanism could be the involvement of a transcriptional repressor of *sox32,* which is positively regulated by Erk phosphorylation.

## Fgf Signaling Is Required for Mesoderm Specification

Our work provides evidence for an essential role for Fgf signaling in the specification of mesoderm. From our immunostainings for P-Smad2 and P-Erk, it is evident that some cells specified as mesoderm receive a combination of Nodal and Fgf signaling, while others, further from the YSL, experience only Fgf signaling (Figure 7B). We previously demonstrated that expression of mesodermal markers, beyond the Nodal signaling domain of five cell tiers, for example *ta*, is induced by Fgf signaling (van Boxtel et al., 2015). We now show that the expression of another crucial transcription factor for mesoderm induction, Noto, is also induced by Fgf signaling in the same domain (Talbot et al., 1995). Similarly, generation of Nodal-expressing clones in the animal pole leads to Fgf signaling-dependent transcription of mesodermal transcription factors *ta* and *noto*. It is likely that the relative levels of Nodal and Fgf signaling within the margin pattern the mesoderm further at later time points, although the details of this remain to be worked out.

## Concluding Remarks

This study reveals the importance of feedforward and feedback loops involving multiple dynamic signaling pathways in spatial patterning, rather than single morphogen gradients. Previous models of mesoderm and endoderm specification have focused on the role of graded Nodal signaling to confer positional information. Our work now demonstrates that it is the interplay between Nodal and downstream Fgf signaling that provides this information in the developing embryo, and determines which

cells are competent to become endoderm progenitors, and which mesoderm. Our proposed mechanism thus explains how graded Nodal signaling can induce two non-overlapping cell fates, and it is the transcription boundary of the inhibitor (Dusp4) that defines the position of the boundary between them. These findings will form the basis for further investigations into embryonic patterning, but also in directed differentiation of embryonic stem cells for use in regenerative medicine. Indeed, in micro-patterned human embryonic stem cell colonies, a ring of *SOX17*-expressing cells is formed within the domain of active NODAL signaling cells, but is mutually exclusive from an inner ring of P-ERK-positive cells that express *T* (Simunovic and Brivanlou, 2017; Warmflash et al., 2014). This suggests that lessons learned from the zebrafish may be directly applicable to mammalian systems.

## STAR★METHODS

Detailed methods are provided in the online version of this paper and include the following:

- KEY RESOURCES TABLE
- CONTACT FOR REAGENT AND RESOURCE SHARING
- EXPERIMENTAL MODEL AND SUBJECT DETAILS
  - Zebrafish Husbandry
- METHOD DETAILS
  - Zebrafish Embryo Culture
  - Chemical Inhibitions
  - Plasmids and mRNA Synthesis
  - Morpholino Injection
  - WISH and Immunohistochemistry
  - Generation of Ndr1-Expressing Clones
  - Image Acquisition
  - Dissociated Zebrafish Embryo Experiments
  - qPCR
  - Western Blotting
  - Smad2 Chromatin Immunoprecipitation (ChIP)
- QUANTIFICATION AND STATISTICAL ANALYSIS
  - Measuring Expression Profiles
  - Quantification of Endodermal Cell Numbers
  - Identification of *dusp4* Regulatory Regions

## SUPPLEMENTAL INFORMATION

Supplemental Information includes six figures and one table and can be found with this article online at https://doi.org/10.1016/j.devcel.2017.11.021.

## ACKNOWLEDGMENTS

We would like to thank Steve Wilson, Anming Meng, and Alex Schier for reagents. We are very grateful to the Francis Crick Institute Aquatics, Light Microscopy, and Experimental Histopathology Facilities. We thank James Briscoe, Nic Tapon, David Wilkinson, and all members of the Hill lab for discussions and useful comments on the manuscript. We thank Marije Schaafsma for help with statistics, and Guillaume Salbreux and Silvia Grigolon for very valuable discussions. This work was supported by the Francis Crick Institute, which receives its core funding from Cancer Research UK (FC001095), the UK Medical Research Council (FC001095), and the Wellcome Trust (FC001095). In addition, this work was supported by the European Commission Network of Excellence EpiGeneSys (HEALTH-F4-2010-257082).

## AUTHOR CONTRIBUTIONS

A.L.v.B. and C.S.H. conceived the study. A.L.v.B., A.D.E., and C.H. designed and performed the experiments. A.L.v.B. and C.S.H. wrote the paper with essential input from A.D.E.

Received: July 20, 2017
Revised: October 20, 2017
Accepted: November 27, 2017
Published: December 21, 2017

## SUPPORTING CITATIONS

The following reference appears in the Supplemental Information: Trinh et al. (2003).

## REFERENCES

Alexander, J., and Stainier, D.Y. (1999). A molecular pathway leading to endoderm formation in zebrafish. Curr. Biol. *9*, 1147–1157.

Anastasaki, C., Rauen, K.A., and Patton, E.E. (2012). Continual low-level MEK inhibition ameliorates cardio-facio-cutaneous phenotypes in zebrafish. Dis. Model. Mech. *5*, 546–552.

Aoki, K., Kumagai, Y., Sakurai, A., Komatsu, N., Fujita, Y., Shionyu, C., and Matsuda, M. (2013). Stochastic ERK activation induced by noise and cell-to-cell propagation regulates cell density-dependent proliferation. Mol. Cell *52*, 529–540.

Bennett, J.T., Joubin, K., Cheng, S., Aanstad, P., Herwig, R., Clark, M., Lehrach, H., and Schier, A.F. (2007). Nodal signaling activates differentiation genes during zebrafish gastrulation. Dev. Biol. *304*, 525–540.

Bisgrove, B.W., Essner, J.J., and Yost, H.J. (1999). Regulation of midline development by antagonism of lefty and nodal signaling. Development *126*, 3253–3262.

Bjornson, C.R., Griffin, K.J., Farr, G.H., 3rd, Terashima, A., Himeda, C., Kikuchi, Y., and Kimelman, D. (2005). Eomesodermin is a localized maternal determinant required for endoderm induction in zebrafish. Dev. Cell *9*, 523–533.

Bogdanovic, O., Fernandez-Minan, A., Tena, J.J., de la Calle-Mustienes, E., Hidalgo, C., van Kruysbergen, I., van Heeringen, S.J., Veenstra, G.J., and Gomez-Skarmeta, J.L. (2012). Dynamics of enhancer chromatin signatures mark the transition from pluripotency to cell specification during embryogenesis. Genome Res. *22*, 2043–2053.

Brown, J.L., Snir, M., Noushmehr, H., Kirby, M., Hong, S.K., Elkahloun, A.G., and Feldman, B. (2008). Transcriptional profiling of endogenous germ layer precursor cells identifies dusp4 as an essential gene in zebrafish endoderm specification. Proc. Natl. Acad. Sci. USA *105*, 12337–12342.

Caunt, C.J., and Keyse, S.M. (2013). Dual-specificity MAP kinase phosphatases (MKPs): shaping the outcome of MAP kinase signalling. FEBS J. *280*, 489–504.

Chen, Y., and Schier, A.F. (2001). The zebrafish Nodal signal Squint functions as a morphogen. Nature *411*, 607–610.

Coda, D.M., Gaarenstroom, T., East, P., Patel, H., Miller, D.S.J., Lobley, A., Matthews, N., Stewart, A., and Hill, C.S. (2017). Distinct modes of SMAD2 chromatin binding and remodeling shape the transcriptional response to NODAL/Activin signaling. Elife *6*, e22474.

Dickmeis, T., Mourrain, P., Saint-Etienne, L., Fischer, N., Aanstad, P., Clark, M., Strahle, U., and Rosa, F. (2001). A crucial component of the endoderm formation pathway, CASANOVA, is encoded by a novel sox-related gene. Genes Dev. *15*, 1487–1492.

Dorey, K., and Amaya, E. (2010). FGF signalling: diverse roles during early vertebrate embryogenesis. Development *137*, 3731–3742.

Du, S., Draper, B.W., Mione, M., Moens, C.B., and Bruce, A. (2012). Differential regulation of epiboly initiation and progression by zebrafish Eomesodermin A. Dev. Biol. *362*, 11–23.

Dubrulle, J., Jordan, B.M., Akhmetova, L., Farrell, J.A., Kim, S.H., Solnica-Krezel, L., and Schier, A.F. (2015). Response to Nodal morphogen gradient is determined by the kinetics of target gene induction. Elife *4*, e05042.

Eswarakumar, V.P., Lax, I., and Schlessinger, J. (2005). Cellular signaling by fibroblast growth factor receptors. Cytokine Growth Factor Rev. *16*, 139–149.

Feldman, B., Gates, M.A., Egan, E.S., Dougan, S.T., Rennebeck, G., Sirotkin, H.I., Schier, A.F., and Talbot, W.S. (1998). Zebrafish organizer development and germ-layer formation require nodal-related signals. Nature *395*, 181–185.

Furthauer, M., Van Celst, J., Thisse, C., and Thisse, B. (2004). Fgf signalling controls the dorsoventral patterning of the zebrafish embryo. Development *131*, 2853–2864.

Gao, S., Alarcon, C., Sapkota, G., Rahman, S., Chen, P.Y., Goerner, N., Macias, M.J., Erdjument-Bromage, H., Tempst, P., and Massague, J. (2009). Ubiquitin ligase Nedd4L targets activated Smad2/3 to limit TGF-β signaling. Mol. Cell *36*, 457–468.

Gritsman, K., Talbot, W.S., and Schier, A.F. (2000). Nodal signaling patterns the organizer. Development *127*, 921–932.

Guan, K.L., and Butch, E. (1995). Isolation and characterization of a novel dual specific phosphatase, HVH2, which selectively dephosphorylates the mitogen-activated protein kinase. J. Biol. Chem. *270*, 7197–7203.

Hagos, E.G., and Dougan, S.T. (2007). Time-dependent patterning of the mesoderm and endoderm by Nodal signals in zebrafish. BMC Dev. Biol. *7*, 22.

Hill, C.S. (2016). Transcriptional control by the SMADs. Cold Spring Harb. Perspect. Biol. *8*, a022079.

Kiecker, C., Bates, T., and Bell, E. (2016). Molecular specification of germ layers in vertebrate embryos. Cell. Mol. Life Sci. *73*, 923–947.

Kiefer, P., Mathieu, M., Mason, I., and Dickson, C. (1996). Secretion and mitogenic activity of zebrafish FGF3 reveal intermediate properties relative to mouse and *Xenopus* homologues. Oncogene *12*, 1503–1511.

Kikuchi, Y., Agathon, A., Alexander, J., Thisse, C., Waldron, S., Yelon, D., Thisse, B., and Stainier, D.Y. (2001). Casanova encodes a novel Sox-related protein necessary and sufficient for early endoderm formation in zebrafish. Genes Dev. *15*, 1493–1505.

Kikuchi, Y., Trinh, L.A., Reiter, J.F., Alexander, J., Yelon, D., and Stainier, D.Y. (2000). The zebrafish bonnie and clyde gene encodes a Mix family homeodomain protein that regulates the generation of endodermal precursors. Genes Dev. *14*, 1279–1289.

Kimmel, C.B., Ballard, W.W., Kimmel, S.R., Ullmann, B., and Schilling, T.F. (1995). Stages of embryonic development of the zebrafish. Dev. Dyn. *203*, 253–310.

Korsensky, L., and Ron, D. (2016). Regulation of FGF signaling: recent insights from studying positive and negative modulators. Semin. Cell Dev. Biol. *53*, 101–114.

Kretzschmar, M., Doody, J., Timokhina, I., and Massague, J. (1999). A mechanism of repression of TGFβ/Smad signaling by oncogenic Ras. Genes Dev. *13*, 804–816.

Liu, Z., Lin, X., Cai, Z., Zhang, Z., Han, C., Jia, S., Meng, A., and Wang, Q. (2011). Global identification of SMAD2 target genes reveals a role for multiple co-regulatory factors in zebrafish early gastrulas. J. Biol. Chem. *286*, 28520–28532.

Mangan, S., and Alon, U. (2003). Structure and function of the feed-forward loop network motif. Proc. Natl. Acad. Sci. USA *100*, 11980–11985.

Mathelier, A., Fornes, O., Arenillas, D.J., Chen, C.Y., Denay, G., Lee, J., Shi, W., Shyr, C., Tan, G., Worsley-Hunt, R., et al. (2016). JASPAR 2016: a major expansion and update of the open-access database of transcription factor binding profiles. Nucleic Acids Res. *44*, D110–D115.

Mathieu, J., Griffin, K., Herbomel, P., Dickmeis, T., Strähle, U., Kimelman, D., Rosa, F.M., and Peyriéras, N. (2004). Nodal and Fgf pathways interact through a positive regulatory loop and synergize to maintain mesodermal cell populations. Development *131*, 629–641.

Mizoguchi, T., Izawa, T., Kuroiwa, A., and Kikuchi, Y. (2006). Fgf signaling negatively regulates Nodal-dependent endoderm induction in zebrafish. Dev. Biol. *300*, 612–622.

Mohammadi, M., McMahon, G., Sun, L., Tang, C., Hirth, P., Yeh, B.K., Hubbard, S.R., and Schlessinger, J. (1997). Structures of the tyrosine kinase domain of fibroblast growth factor receptor in complex with inhibitors. Science *276*, 955–960.

Molina, G.A., Watkins, S.C., and Tsang, M. (2007). Generation of FGF reporter transgenic zebrafish and their utility in chemical screens. BMC Dev. Biol. *7*, 62.

Nelson, A.C., Cutty, S.J., Gasiunas, S.N., Deplae, I., Stemple, D.L., and Wardle, F.C. (2017). In vivo regulation of the zebrafish endoderm progenitor Niche by T-box transcription factors. Cell Rep. *19*, 2782–2795.

Nelson, A.C., Cutty, S.J., Niini, M., Stemple, D.L., Flicek, P., Houart, C., Bruce, A.E., and Wardle, F.C. (2014). Global identification of Smad2 and Eomesodermin targets in zebrafish identifies a conserved transcriptional network in mesendoderm and a novel role for Eomesodermin in repression of ectodermal gene expression. BMC Biol. *12*, 81.

Ober, E.A., Field, H.A., and Stainier, D.Y. (2003). From endoderm formation to liver and pancreas development in zebrafish. Mech. Dev. *120*, 5–18.

Ornitz, D.M., and Itoh, N. (2015). The fibroblast growth factor signaling pathway. Wiley Interdiscip. Rev. Dev. Biol. *4*, 215–266.

Poulain, M., Furthauer, M., Thisse, B., Thisse, C., and Lepage, T. (2006). Zebrafish endoderm formation is regulated by combinatorial Nodal, FGF and BMP signalling. Development *133*, 2189–2200.

Poulain, M., and Lepage, T. (2002). Mezzo, a paired-like homeobox protein is an immediate target of Nodal signalling and regulates endoderm specification in zebrafish. Development *129*, 4901–4914.

Reiter, J.F., Kikuchi, Y., and Stainier, D.Y. (2001). Multiple roles for Gata5 in zebrafish endoderm formation. Development *128*, 125–135.

Rodaway, A., Takeda, H., Koshida, S., Broadbent, J., Price, B., Smith, J.C., Patient, R., and Holder, N. (1999). Induction of the mesendoderm in the zebrafish germ ring by yolk cell-derived TGF-β family signals and discrimination of mesoderm and endoderm by FGF. Development *126*, 3067–3078.

Sampath, K., Rubinstein, A.L., Cheng, A.M., Liang, J.O., Fekany, K., Solnica-Krezel, L., Korzh, V., Halpern, M.E., and Wright, C.V. (1998). Induction of the zebrafish ventral brain and floorplate requires cyclops/nodal signalling. Nature *395*, 185–189.

Schier, A.F. (2009). Nodal morphogens. Cold Spring Harb. Perspect. Biol. *1*, a003459.

Schier, A.F., and Talbot, W.S. (2005). Molecular genetics of axis formation in zebrafish. Annu. Rev. Genet. *39*, 561–613.

Schneider, C.A., Rasband, W.S., and Eliceiri, K.W. (2012). NIH Image to ImageJ: 25 years of image analysis. Nat. Methods *9*, 671–675.

Schulte-Merker, S., Ho, R.K., Herrmann, B.G., and Nusslein-Volhard, C. (1992). The protein product of the zebrafish homologue of the mouse T gene is expressed in nuclei of the germ ring and the notochord of the early embryo. Development *116*, 1021–1032.

Schulte-Merker, S., van Eeden, F.J., Halpern, M.E., Kimmel, C.B., and Nusslein-Volhard, C. (1994). No tail (ntl) is the zebrafish homologue of the mouse T (Brachyury) gene. Development *120*, 1009–1015.

Simunovic, M., and Brivanlou, A.H. (2017). Embryoids, organoids and gastruloids: new approaches to understanding embryogenesis. Development *144*, 976–985.

Tabar, V., and Studer, L. (2014). Pluripotent stem cells in regenerative medicine: challenges and recent progress. Nat. Rev. Genet. *15*, 82–92.

Talbot, W.S., Trevarrow, B., Halpern, M.E., Melby, A.E., Farr, G., Postlethwait, J.H., Jowett, T., Kimmel, C.B., and Kimelman, D. (1995). A homeobox gene essential for zebrafish notochord development. Nature *378*, 150–157.

Thisse, B., and Thisse, C. (2005). Functions and regulations of fibroblast growth factor signaling during embryonic development. Dev. Biol. *287*, 390–402.

Trinh, L.A., Meyer, D., and Stainier, D.Y. (2003). The Mix family homeodomain gene bonnie and clyde functions with other components of the Nodal signaling

pathway to regulate neural patterning in zebrafish. Development *130*, 4989–4998.

Tsang, M., Maegawa, S., Kiang, A., Habas, R., Weinberg, E., and Dawid, I.B. (2004). A role for MKP3 in axial patterning of the zebrafish embryo. Development *131*, 2769–2779.

van Boxtel, A.L., Chesebro, J.E., Heliot, C., Ramel, M.C., Stone, R.K., and Hill, C.S. (2015). A temporal window for signal activation dictates the dimensions of a nodal signaling domain. Dev. Cell *35*, 175–185.

Wardle, F.C., and Papaioannou, V.E. (2008). Teasing out T-box targets in early mesoderm. Curr. Opin. Genet. Dev. *18*, 418–425.

Warga, R.M., and Nusslein-Volhard, C. (1999). Origin and development of the zebrafish endoderm. Development *126*, 827–838.

Warmflash, A., Sorre, B., Etoc, F., Siggia, E.D., and Brivanlou, A.H. (2014). A method to recapitulate early embryonic spatial patterning in human embryonic stem cells. Nat. Methods *11*, 847–854.

Westerfield, M. (2000). The Zebrafish Book. A Guide for the Laboratory Use of Zebrafish (*Danio rerio*), Fourth Edition (University of Oregon Press).

Wu, M.Y., and Hill, C.S. (2009). TGF-beta superfamily signaling in embryonic development and homeostasis. Dev. Cell *16*, 329–343.

Xu, P.F., Houssin, N., Ferri-Lagneau, K.F., Thisse, B., and Thisse, C. (2014). Construction of a vertebrate embryo from two opposing morphogen gradients. Science *344*, 87–89.

Zorn, A.M., and Wells, J.M. (2009). Vertebrate endoderm development and organ formation. Annu. Rev. Cell Dev. Biol. *25*, 221–251.

# STAR★METHODS

## KEY RESOURCES TABLE

| REAGENT or RESOURCE | SOURCE | IDENTIFIER |
|---|---|---|
| Antibodies | | |
| Anti-phospho-Smad2 (IF) | Cell Signaling Technology | Cat# 8828; RRID: AB_2631089 |
| Anti-phospho-Erk (IF and Western) | Sigma | Cat# M8159; RRID: AB_477245 |
| Anti-Digoxigenin-AP (*in situ*) | Roche | Cat# 11093274910; RRID: AB_514497 |
| Anti-Digoxigenin-HRP (*in situ*) | Roche | Cat# 1207733910 |
| Anti-DNP-HRP (*in situ*) | Perkin Elmer | Cat# FP1129; RRID: AB_2629439 |
| Anti-DNP-AP (*in situ*) | Vector Laboratories | Cat# MB-3100; RRID: AB_2336089 |
| Anti-Rabbit Alexa Fluor 488 (IF) | Thermo Fisher Scientific | Cat# A-21206; RRID: AB_2535792 |
| Anti-Mouse Alexa Fluor 546 (IF) | Thermo Fisher Scientific | Cat# A-10036; RRID: AB_2534012 |
| Anti-Mouse Alexa Fluor 594 (IF) | Thermo Fisher Scientific | Cat# A-21203; RRID: AB_2535789 |
| Anti-Rabbit Alexa Fluor 647 (IF) | Thermo Fisher Scientific | Cat# A-21244; RRID: AB_10562581 |
| Anti-Mouse-HRP (Western) | Dako | Cat# P0447; RRID: AB_2617137 |
| Anti-Rabbit-HRP (Western) | Dako | Cat# P0448; RRID: AB_2617138 |
| Anti-Erk1 (Western) | Santa Cruz | Cat# sc-94; RRID: AB_2140110 |
| Anti-Actin (Western) | Sigma | Cat# A3853; RRID: AB_262137 |
| Anti-Smad2 (31H15L4) (ChIP) | Thermo Fisher Scientific | Cat# 700048; RRID: AB_2532277 |
| Anti-Smad2 (D43B4) (ChIP) | Cell Signaling Technology | Cat# 5339; RRID: AB_10626777 |
| Chemicals, Peptides, and Recombinant Proteins | | |
| SB-505124 | Sigma | Cat# S4696 |
| SU-5402 | Merck | Cat# 572631 |
| PD-0325901 | Merck | Cat# 444968 |
| NBT/BCIP tablets | Sigma | Cat# B5655 |
| Fast Red TR/Naphthol AS-MX Tablets | Sigma | Cat# F4648 |
| Tyramine hydrochloride | Sigma | Cat# T2879 |
| NHS-Fluorescein ester | Thermo Fisher Scientific | Cat# 46410 |
| Cy5 Mono NHS ester | Sigma | Cat# PA51501 |
| Human recombinant NODAL | R&D | Cat# 3218-ND/CF |
| Human basic FGF | Peprotech | Cat# AF100-18b |
| Critical Commercial Assays | | |
| QuikChange XL Site-Directed Mutagenesis Kit | Agilent | Cat# 200516 |
| Experimental Models: Organisms/Strains | | |
| Zebrafish *Danio rerio*: WT | N/A | N/A |
| Oligonucleotides | | |
| See Table S1 for list of all primers, cloning oligonucleotides and morpholinos | This study | N/A |
| Recombinant DNA | | |
| pBKS-*ntl-a*: probe synthesis *ta*: linearize Xho1: polymerase T7 | Schulte-Merker et al., 1992 | N/A |
| pAD-gal4-*Lft2*: probe synthesis *lft2*: linearize Mlu1: polymerase T7 | Bisgrove et al., 1999 | N/A |
| pBSK-*flh*: probe synthesis *noto*: linearize EcoR1: polymerase T7 | Talbot et al., 1995 | N/A |
| pBSK-*fgf3*: probe synthesis *fgf3*: linearize BamH1: polymerase T7 | Kiefer et al., 1996 | N/A |

(*Continued on next page*)

| Continued | | |
|---|---|---|
| REAGENT or RESOURCE | SOURCE | IDENTIFIER |
| pBSK-*fgf8a* (cb110): probe synthesis *fgf8a*: linearize Not1: polymerase T7 | https://zfin.org/ZDB-PUB-010810-1 ZFIN online publication | N/A |
| pBKS-*sox17*: probe synthesis *sox17*: linearize Nco1: polymerase Sp6 | Alexander and Stainier, 1999 | N/A |
| pBS-*sox32* (cb527): probe synthesis *sox32*: linearize Not1: polymerase T7 | https://zfin.org/ZDB-PUB-010810-1 ZFIN online publication | N/A |
| pCS2+*ndr1*: mRNA synthesis *ndr1*: linearize Not1: polymerase Sp6 | Feldman et al., 1998 | N/A |
| pCS2-*dusp4*: mRNA synthesis *dusp4*: linearize Not1: polymerase Sp6 | This study | N/A |
| pCS2-*dusp4*_ATG-mut: mRNA synthesis mutant *dusp4*: linearize Not1: polymerase Sp6 | This study | N/A |
| pCS2-*fgf8a:* mRNA synthesis *fgf8a*: linearize Not1: polymerase Sp6 | van Boxtel et al., 2015 | N/A |
| Software and Algorithms | | |
| FIJI (ImageJ) | Schneider et al., 2012 | https://imagej.net/Fiji/Downloads |
| JASPAR | Mathelier et al., 2016 | http://jaspar.genereg.net/ |

## CONTACT FOR REAGENT AND RESOURCE SHARING

Further information and requests for resources and reagents should be directed to and will be fulfilled by the Lead Contact, Caroline Hill (caroline.hill@crick.ac.uk).

## EXPERIMENTAL MODEL AND SUBJECT DETAILS

### Zebrafish Husbandry

Wild type zebrafish (*Danio rerio*) were maintained under standard conditions (Westerfield, 2000). Adult zebrafish were kept on a regular light-dark cycle (14 hours on/10 hours off) at 27°C. Note, analysis of early embryos precludes determination of animal sex.

All the zebrafish work was carried out under a UK Home Office License under the Animals (Scientific Procedures) Act 1986. The license underwent full ethical review and approval by the Francis Crick Institute's Animal Ethics Committee.

## METHOD DETAILS

### Zebrafish Embryo Culture

All experiments in live embryos were performed at 28°C. Embryos were carefully staged according to morphological features and where needed for time courses, collected in a 5 min interval after removing dividers from breeding tanks to ensure synchronization (Kimmel et al., 1995). Embryos were fixed in 4% paraformaldehyde overnight at 4°C, dechorionated, dehydrated to 100% methanol and stored at -20°C until processing.

### Chemical Inhibitions

The inhibitors SU-5402 (Calbiochem, #572631), PD-0325901 (Merck, #444968) and SB-505124 (Sigma, #S4696) were dissolved in DMSO and directly diluted in embryo medium at 10 μM, 5 μM and 50 μM respectively.

### Plasmids and mRNA Synthesis

The zebrafish *dusp4* open reading frame (ORF) was cloned by PCR amplification using pooled blastula stage cDNA with oligonucleotides elongated with BamH1/Xho1 sites that were used to clone the fragment into the pCS2+ plasmid. A version of pCS2+-*dusp4* was also generated for rescue of the translation blocking MO (MO1) in which five silent point mutations were introduced in the first 25 base pairs of the *dusp4* ORF, using the Agilent QuikChange XL kit according to the manufacturer's instructions. Primer sequences are given in Table S1. For full length capped mRNA, the pCS2+-*ndr1*, pCS2-*Fgf8a* and pCS2+-*dusp4* plasmids were linearized using Not1 (NEB) and transcribed using SP6 RNA polymerase (NEB) for 2–3 hrs. Template DNA was removed using DNAseI (Worthington) treatment for 30 min at 37°C and mRNA was subsequently purified by Lithium Chloride extraction, reconstituted in water and stored at -80°C until injection (Gritsman et al., 2000; van Boxtel et al., 2015).

## Morpholino Injection

For a full list of MOs (Genetools), references, their use and dilutions, see Table S1. MOs were diluted in $H_2O$ and injected in 1–2 cell stage embryos in a volume of 2 nl. The efficacy of each MO was determined by injecting a range of concentrations and effective concentrations ranged from 3–8 ng per embryo. For *dusp4*, a start site MO (MO1) was designed and two splice site MOs (MO2 and MO3) were used that have previously been characterized (Brown et al., 2008). For both MO2 and MO3, knockdown was determined by RT-PCR in multiple experiments. For the MO rescue experiments, full length capped wild type *dusp4* mRNA was co-injected with the MO2 and MO3 at 400 pg per embryo. For rescue of MO1, the mutated version of *dusp4* mRNA was used (see above). The *dusp6* MO1 has been characterized previously (Tsang et al., 2004) and efficacy of the splice site *dusp6* MO (MO2) was determined by RT-PCR.

## WISH and Immunohistochemistry

All plasmids for the generation of riboprobes, with references can be found in the Key Resources Table. For probes that were generated from PCR products, see Table S1. Standard WISH, including the addition of 5% dextran sulphate to the hybridization buffer, was performed as described (van Boxtel et al., 2015). In brief, samples were rehydrated to PBS/0.1% Tween (PTW) before hybridization with digoxigenin (Dig)-11-UTP- (Roche, #11209256910) labeled riboprobes against the indicated target genes, overnight at 65°C. Embryos were then incubated overnight at 4°C with anti-Dig-AP (Roche, #11093274910; 1:5000). Embryos were washed extensively in PTW before detecting alkaline phosphatase with NBT/BCIP (Sigma, # B5655).

For FISH, samples were first incubated in 2% $H_2O_2$ in 100% methanol for 20 min to reduce background staining before rehydration to PTW. Hybridization with an additional riboprobe labeled with dinitrophenol (DNP)-11-UTP (Perkin Elmer, #NEL555001EA) allowed two targets to be visualized simultaneously. Embryos were then incubated overnight at 4°C with anti-Dig-AP as for standard WISH, or anti-Dig-HRP (Roche, #1207733910, 1:500), anti-DNP-HRP (Perkin Elmer, #FP1129) or anti-DNP-AP (Vector labs MB-3100, 1:1000) antibodies, followed by extensive washes in PTW. To detect HRP, embryos were incubated with tyramide (Sigma, #T2879) coupled to either fluorescein-NHS ester (Thermo Scientific, #46410) or Cy5 mono NHS ester (Sigma, #PA15101) for 25 min in the dark in PTW. Following the addition of 0.001% $H_2O_2$ signal was allowed to develop for 30 min. After two washes with PTW, Fast Red (Sigma, #F4648) was used according to the manufacturer's instructions to detect the AP. The embryos were then extensively washed in PTW, and DAPI was used at 1:5000 as a nuclear counter stain.

For all FISH and regular WISH experiments, the number of representative embryos out of the total number of embryos stained, is depicted in the right bottom corner of each image. Sectioning of standard WISH-stained embryos was performed as previously described (van Boxtel et al., 2015). Embryos were embedded in paraffin, sectioned at 8 μm and counterstained with Nuclear Fast Red for 5 min (Vector laboratories, H3403).

Immunohistochemistry was performed as described (van Boxtel et al., 2015). Embryos were rehydrated, washed extensively in PBS/1% Triton X-100 and incubated in cold acetone at -20°C for 20 min, before blocking in 10% FBS and 1% Triton X-100 in PBS. Embryos were incubated with antibodies against P-Smad2 (Cell Signaling Technology, #8828, 1:1000) and P-Erk (Sigma, #M8159, 1:500) at 4°C overnight. For visualization, the following secondary antibodies were used at 1:500: donkey anti-rabbit Alexa Fluor 488, donkey anti-mouse Alexa Fluor 594, donkey anti-mouse Alexa Fluor 546, goat anti-rabbit Alexa Fluor 647 (Thermofisher), and DAPI as a nuclear counter stain.

## Generation of Ndr1-Expressing Clones

To generate clones of Ndr1-expressing cells, one- to two-cell stage embryos were either untreated or were injected with *dusp4* mRNA and raised until the 64- to 128-cell stage. At these stages, a single blastomere was injected with 1% dextran- fluorescein to allow Ndr1-expressing cells to be visualized and 10 pg capped *ndr1* mRNA under a stereomicroscope. The injected embryos were allowed to recover in E3 medium in agarose-coated dishes and either left untreated or treated with DMSO or 5 μM PD-0325901 from sphere stage, and then collected at germ ring stage for immunohistochemistry or FISH. Experiments were performed at least in duplicate and a minimum of four embryos was analyzed for each condition.

## Image Acquisition

All imaging for FISH-stained embryos and immuno-stained embryos was performed using Zeiss LSM710, 780 or 880 confocal microscopes. To this end, embryos were either mounted in 0.8% low melt agarose on 35 mm glass bottom dishes (Matek, P35G-1.5-14C) to image the lateral margin, or the entire margin was imaged by dissecting the yolk from the embryo which was then flat-mounted in Mowiol (Calbiochem, #475904). Whole-mounted embryos were imaged with a 25×/0.8 LD LCI Plan-Apochromat water immersion lens, and flat-mounted embryos were imaged with a 10×/0.45 Ph1 Plan-Apochromat lens. For illustrative purposes, maximum or average intensity Z-projections from confocal stacks were generated and adjusted to enhance contrast and brightness where appropriate. Further adjustments were performed using Gaussian blur with a radius of one pixel and within experiments, adjustments were kept equal between control and treated samples. For Z-reconstructions specimens were mounted in 90% glycerol in 0.1 M Tris HCl pH 8.5 and imaged with a 25×/0.8 LD LCI Plan-Apochromat oil immersion lens. Light sheet imaging was performed using a Luxendo MuVi-SPIM, with resulting image stacks resliced to give the appropriate orientation.

## Dissociated Zebrafish Embryo Experiments

Zebrafish embryonic cell culture experiments were performed at 28°C with pre-warmed buffers (van Boxtel et al., 2015). Blastomeres were obtained from up to 1000 high–oblong stage embryos that were dechorionated in 2 mg/ml Pronase (Roche, #10165921001) in E3 medium, washed extensively in E3 to remove remaining Pronase and equilibrated in Calcium free Ringers buffer, before gently dissociating cells using a P200 pipette in a volume of 10 ml. The cells were collected by centrifugation for 5 min at 1000 x g and the pellet was gently disrupted before resuspension in Leibovitz's L15 medium (Gibco, #11415-064) without serum, at a density of 50 embryos/ml and seeded on 24 well tissue culture plates coated with poly-L lysine in a final volume of 1 ml. Human recombinant NODAL (R&D, #3218-ND/CF) was dissolved in 4 mM HCl at 100 mg/ml, aliquoted in non-stick tubes, stored at -80°C and used at indicated concentrations without freeze-thawing. Human basic FGF (Peprotech, #AF100-18b) was dissolved according to the manufacturer's instructions and dilutions of both recombinant proteins were made directly in serum free L15 medium in non-stick tubes. Seeded cells were incubated with the indicated concentrations of ligands for 2 hrs after which the medium was aspirated and the cells snap-frozen at -80°C until processing for qPCR. As controls, Western blotting was performed for P-Smad2 and P-Erk in parallel.

## qPCR

For qPCR on whole embryos, 5–10 embryos were snap frozen in a minimal amount of medium without dechorionation. qPCR was performed as previously described (van Boxtel et al., 2015) and for primer sequences see Table S1. In brief, mRNA was extracted using Trizol (Thermo Fisher Scientific) and cDNA synthesis was performed on 500 ng mRNA using Affinityscript (Qiagen), both according to the manufacturer's instructions. qPCRs were performed using Fast SYBR Green Master mix (Thermo Fisher Scientific) on an ABI 7500 Fast (Applied Biosystems) thermocycler. For each primer set, qPCR efficiencies and specificity were first determined using standard curves of diluted cDNAs and melting curve analysis. Technical replicates for each condition were taken and experiments were repeated five times. Caculations were performed using the ΔΔCt method. Means ± SEM from at least two independent experiments are shown. Statistics were performed on these data using a t test.

## Western Blotting

Western blotting was performed as previously described (van Boxtel et al., 2015). Five to ten embryos were snap frozen and stored at -80°C until processing. Lysates were generated by homogenizing the pooled embryos in lysis buffer (10 μl per embryo; 20 mM Tris HCl pH 8, 2 mM EDTA pH 8, 0.5% NP-40, 25 mM β-glycerophosphate, 100 mM NaF, 20 nM Calyculin A, 100 mM sodium pyrophosphate and protease inhibitors). The equivalent of 1–2 embryos was loaded onto standard 15% SDS polyacrylamide gels. After electrophoresis, proteins were transferred to PDVF membrane (Millipore) and immunoblotted using standard techniques. The following antibodies were used: anti-phosphorylated-Erk (Sigma, #M8159), anti-Erk (Santa Cruz, #sc-94) and anti-Actin (Sigma, #A3853).

## Smad2 Chromatin Immunoprecipitation (ChIP)

Chromatin immunoprecipitation (ChIP) assays were performed using 50% epiboly embryos treated from sphere stage with DMSO and SB-505124. Chromatin was prepared and sheared to a range of 0.3 to 0.7 kb by sonication and the equivalent of 70 μg of chromatin was used in each ChIP experiment and immunoprecipitated with a mix of rabbit anti-Smad2 (Thermo Fisher, #700048) and rabbit anti-Smad2 (Cell Signaling technologies, #5339) antibodies (Coda et al., 2017; Nelson et al., 2014). Individual input dilutions corresponding to treatments were used as qPCR standard curves to quantitate Smad2 binding in the corresponding region and these values were then normalized using the *actin* negative control region. As a positive control, we used a known Smad2-binding enhancer associated with *mixl1* (Nelson et al., 2014) and normalization was validated with the *sox10* TSS as a negative control region. For all oligonucleotides used in these experiments, see Table S1.

# QUANTIFICATION AND STATISTICAL ANALYSIS

## Measuring Expression Profiles

Quantification of nuclear P-Smad2 and P-Erk intensity, relative to the margin on whole-mounted embryos was carried out as previously described (van Boxtel et al., 2015), but with modifications to allow semi-automation. Using ImageJ software (Schneider et al., 2012) individual nuclei were segmented from the DAPI staining of each optical slice by an automated threshold followed by binary water shedding. To avoid selecting mitotic figures, overlapping nuclei or small nuclear extremities, nuclei with a cross sectional area greater than 50 pixels (8.6 $\mu m^2$) and circularity greater than 0.75 were selected. A region of interest (ROI) was drawn on each optical section to exclude nuclei from the enveloping layer (EVL) and YSL. Selected nuclei were used as ROIs to measure average DAPI intensity, as well as P-Smad2 and P-Erk intensity, and allowing P-Smad2 or P-Erk to DAPI ratios to be calculated. This was carried out on at least three optical sections per embryo. To measure the distance of each nucleus to the margin boundary, points along this boundary were marked out on a maximum projection of each embryo and a spline curve fitted using ImageJ software (Schneider et al., 2012), with the distance to the boundary taken as the minimum distance from each nuclear centroid to the boundary curve. For each time point the data were divided into 15 μm bins and within each bin weighted means of the normalized intensities across at least three embryos were calculated, as was the normalized standard deviation (as indicated in Figure legends).

To quantify intensity profiles around the margin for flat-mounted embryos stained by immunohistochemistry or FISH (for P-Smad2, P-Erk, *noto, ta, dusp4* and *lft2)*, the border between the YSL and embryonic margin was marked out on maximum projections and a spline curve fitted. At pixel intervals a line perpendicular to this boundary was calculated, and the intensity profile along this line

150 μm (approximately 10 cell tiers) into the embryo was measured. From this, an average profile around the margin was calculated by taking the mean intensities at pixel increments away from the boundary. The represented data show means of traces from at least three different embryos per condition, and their SD (as indicated in Figure legends).

### Quantification of Endodermal Cell Numbers

To determine endodermal cell numbers at 75% epiboly, embryos were stained by standard WISH for *sox17*, dehydrated in methanol and cleared in 80% glycerol. To visualize and count endodermal progenitors, the yolk was removed and the blastoderm flat-mounted on histological slides, imaged under a stereo microscope (Leica MZ8) after which endodermal progenitors were counted manually. Care was taken to exclude dorsal forerunner cells. For quantifications of endodermal cell numbers at 50% epiboly, embryos were stained for *lft2* and *sox32*, flat-mounted and imaged under a confocal microscope (as described above) to generate Z-stacks with an interval of 2–3 μm. *sox32*-positive endodermal cells were counted manually only recording cells positive cells for both *sox32* and *lft2* to exclude dorsal forerunner cells and *sox32* staining in the YSL. In initial experiments, these counts were done blind. For all experiments where cell numbers were counted, data are represented as an open red circle. Statistics were performed on these data using a Mann-Whitney U test (as indicated in Figure legends). For quantifications of endodermal cell numbers surrounding the Ndr1-expressing clones, *sox32*-positive cells were counted manually using the Z-Stacks. The surface area of *lft2* expression domains was measured using Image J software over at least 11 embryos from three independent experiments. Statistics were performed on these data using a two-tailed t test.

### Identification of *dusp4* Regulatory Regions

To confirm that *dusp4* is a direct Nodal target, genomic regulatory regions associated with *dusp4* that bound Smad2 were identified. To this end, three published datasets were used (Dubrulle et al., 2015; Liu et al., 2011; Nelson et al., 2014), and two of these datasets identified *dusp4* as a direct Nodal target gene. To identify all regions with potential Smad binding sites, enhancers marked by H3K4me1 and H3K27Ac at dome-stage (Bogdanovic et al., 2012) were located and scanned for a FoxH1–Smad2–Smad4 motif using JASPAR (Mathelier et al., 2016). Using these criteria, two potential enhancer regions were identified, located at -31.3kb and -3.2kb from the *dusp4* TSS.

**Developmental Cell, Volume *44***

## Supplemental Information

# Long-Range Signaling Activation and Local Inhibition Separate the Mesoderm and Endoderm Lineages

**Antonius L. van Boxtel, Andrew D. Economou, Claire Heliot, and Caroline S. Hill**

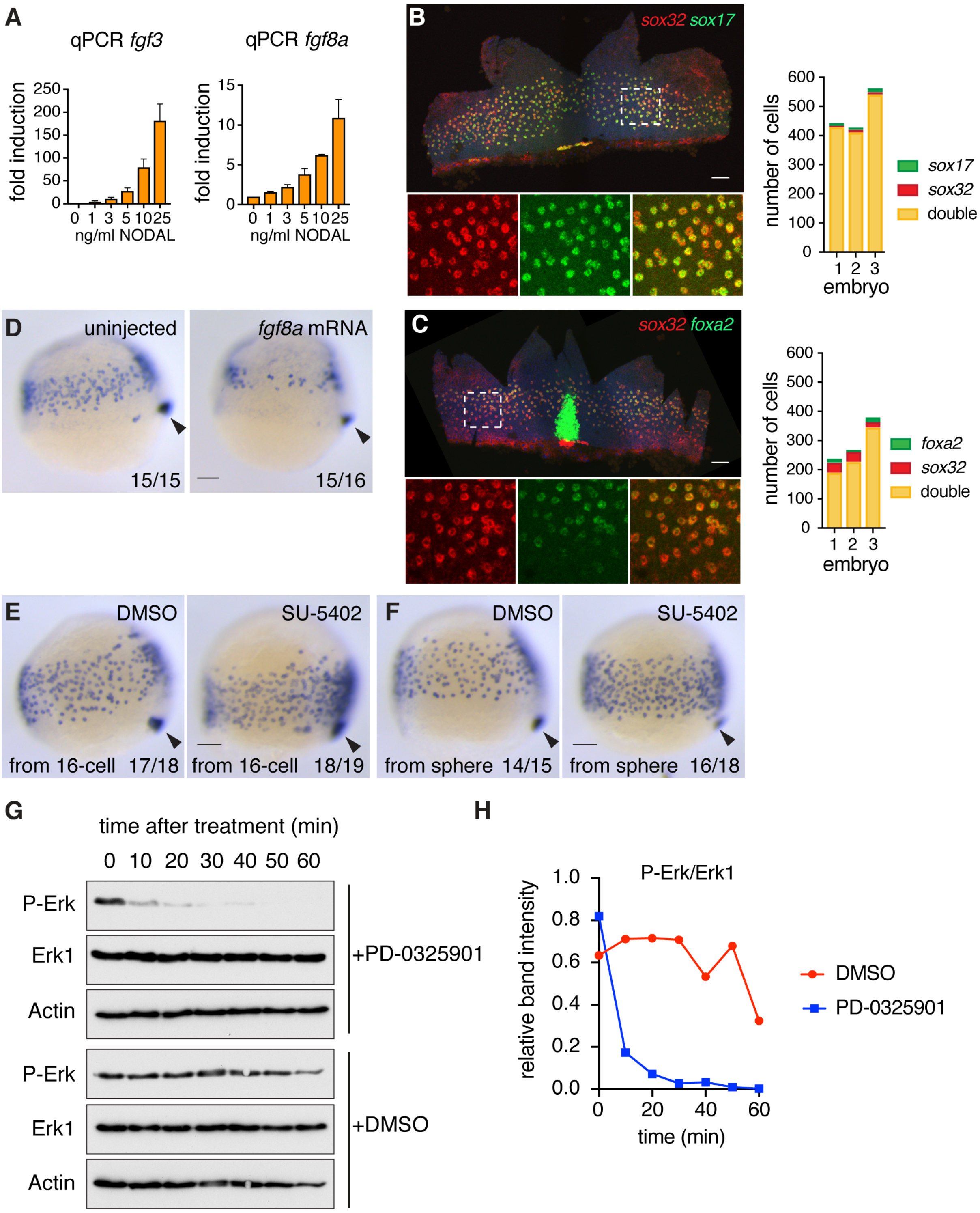


**Figure S1, related to Figure 1. Fgf signaling inhibits endoderm specification**

(A) qPCR for *fgf3* and *fgf8a* in zebrafish dissociated embryos treated with increasing concentrations of NODAL, mean ± SEM, n >3.

(B) Left panel: Flat-mounted 80% epiboly embryo stained for *sox32* and *sox17*. Box with dashed white line indicates the enlarged area presented in the bottom panels where the staining

is shown for the individual genes and the two combined. Right panel: quantification of endoderm progenitors from three embryos stained as in left panel.

(C) As in (B) but for *sox32* and *foxa2* in 70% epiboly embryos. The strong *foxa2* staining in the center of the flat-mount image corresponds to the *foxa2* expressing axial mesoderm.

(D) Representative images of either uninjected or *fgf8a* mRNA-injected embryos (10 pg) isolated at 75% epiboly and stained for *sox17* by WISH. Overexpression of Fgf8a results in reduced endodermal cell numbers. Arrowhead, dorsal forerunner cells, which mark the most posterior end of the embryo to compare staging.

(E) Representative images of 75% epiboly embryos stained for *sox17* by WISH after treatment with either DMSO as control or 10 µM SU-5402. Chemicals were added from the 16-cell stage. Arrowhead, dorsal forerunner cells. Note that as well as increasing *sox17*-positive endoderm progenitors, SU-5402 also partially inhibits cell migration.

(F) Same as in (E) but for embryos treated from sphere stage. Note that Fgf signaling inhibition in (E) and (F) both result in more endodermal progenitors.

(G) 50% epiboly embryos were treated with DMSO or PD-0325901 for the times shown. Whole cell extracts were Western blotted for P-Erk, Erk1 and Actin as a loading control.

(H) The Western blots in (G) were scanned and quantitated using ImageJ.

All scale bars are 100 µm.

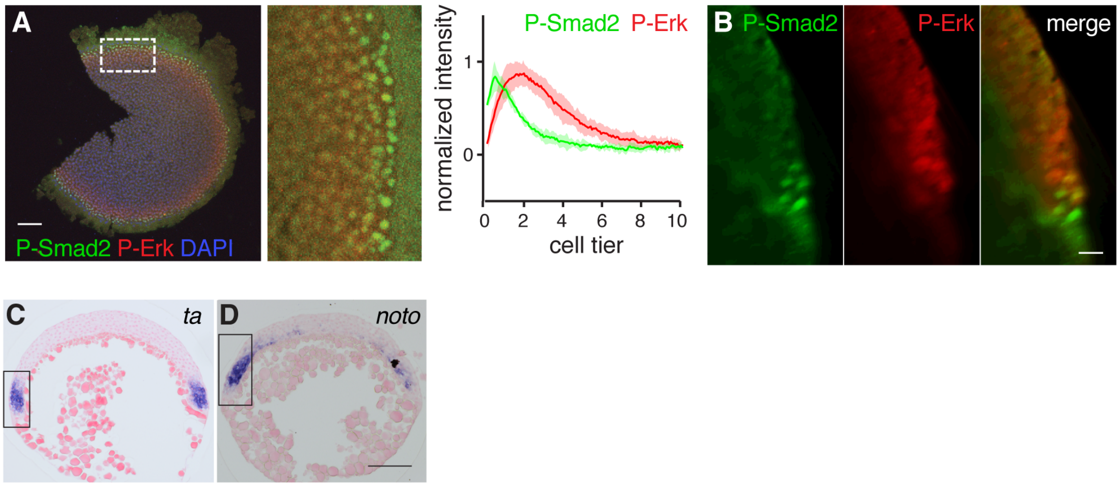


**Figure S2, related to Figure 2. Cells directly adjacent to the YSL maintain low levels of P-Erk**

(A) Example of flat-mounted, 50% epiboly embryo, stained by immunohistochemistry for P-Erk and P-Smad2. Right panel shows average of intensity traces of three embryos (mean ± SD). Scale bar, 100 µm.

(B) Resliced optical section of 50% epiboly embryo stained as in (A) obtained with light sheet microscope. Note that nuclei close to the YSL have high P-Smad2, but low P-Erk levels. Scale bar, 25 µm.

(C) Section of paraffin embedded WISH-stained 50% embryo for *ta*. Dorsal to the right. Box indicates enlarged area in Figure 2G. Scale bar, 100 µm.

(D) As in (C) for *noto,* except that dorsal is to the left.

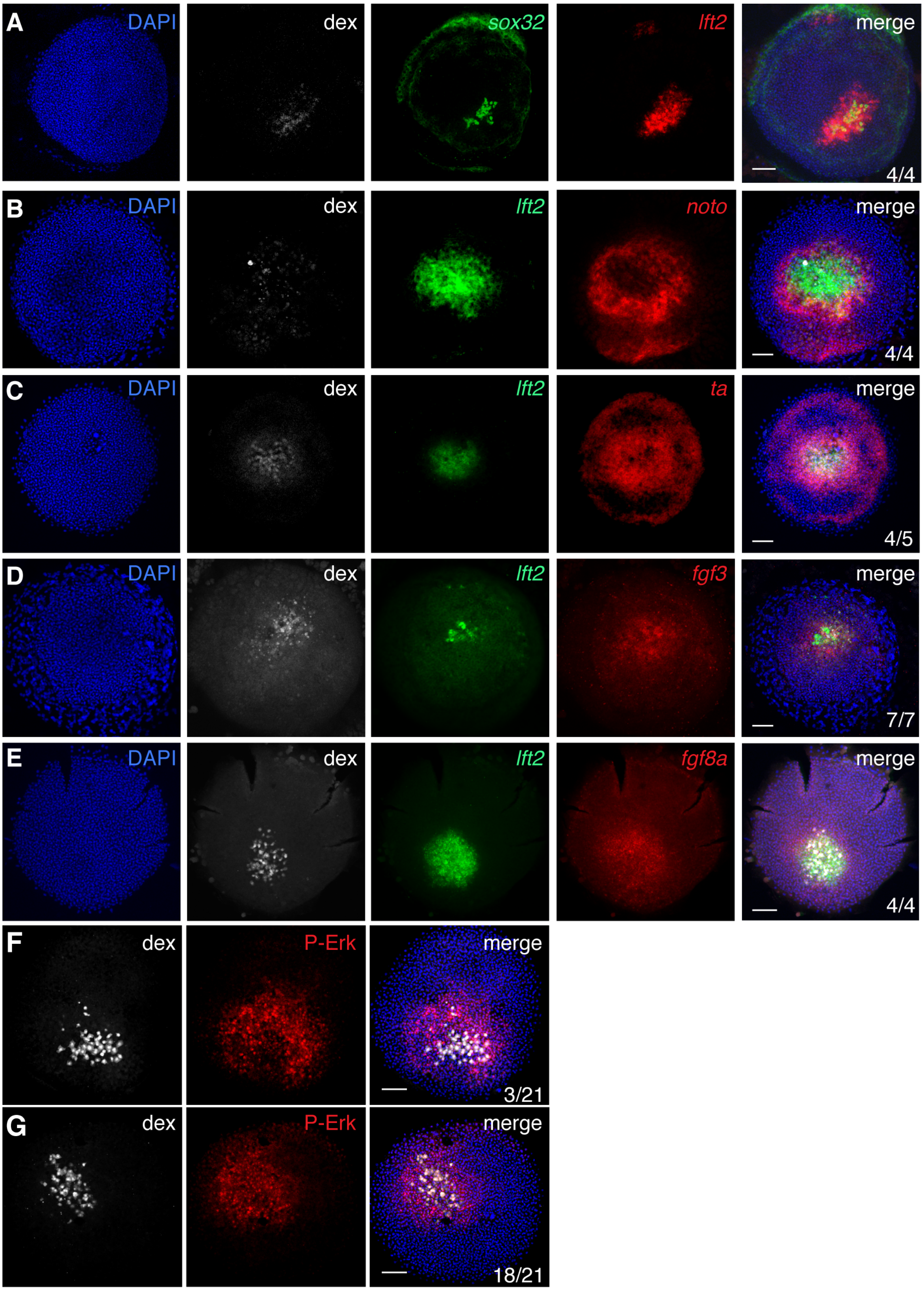


**Figure S3, related to Figure 3. Nodal-induced patterning in the animal pole mimics patterning in the margin**

(A) Flat-mounted germ ring stage embryos containing a Nodal-expressing clone stained for *sox32* and *lft2*. This is the same embryo shown in Figure 3B left panel, but here the individual

channels are shown. The Nodal-expressing clones are visualized with the dextran fluorescein (dex), and nuclei are visualized with DAPI. A merge of all channels is also shown. Note that *sox32*-positive cells are specified within the *lft2*-positive domain. In the bottom right corner, the number of representative embryos is depicted out of total analyzed embryos from two independent experiments.

(B) As in (A) but for *lft2* and *noto*. *noto* expression is lower in the *lft2* expressing domain. This is the same embryo shown in Figure 3B, middle panel.

(C) As in (A) but for *lft2* and *ta.* Expression of *ta* is lower within the *lft2* expressing domain. This is the same embryo shown in Figure 3B, right panel.

(D) As in (A) but for *lft2* and *fgf3.* This is the same embryo shown in Figure 3C, left panel.

(E) As in (A) but for *lft2* and *fgf8a.* This is the same embryo shown in Figure 3C, right panel.

(F and G) Examples of Nodal-expressing clones visualized with dextran fluorescein (dex) and stained for P-Erk. In very dense clones (3/21) it is clear that the cells expressing Nodal show a clear domain of low P-Erk staining (F). In other clones (18/21), the P-Erk staining is heterogeneous throughout (G).

All scale bars are 100 µm.

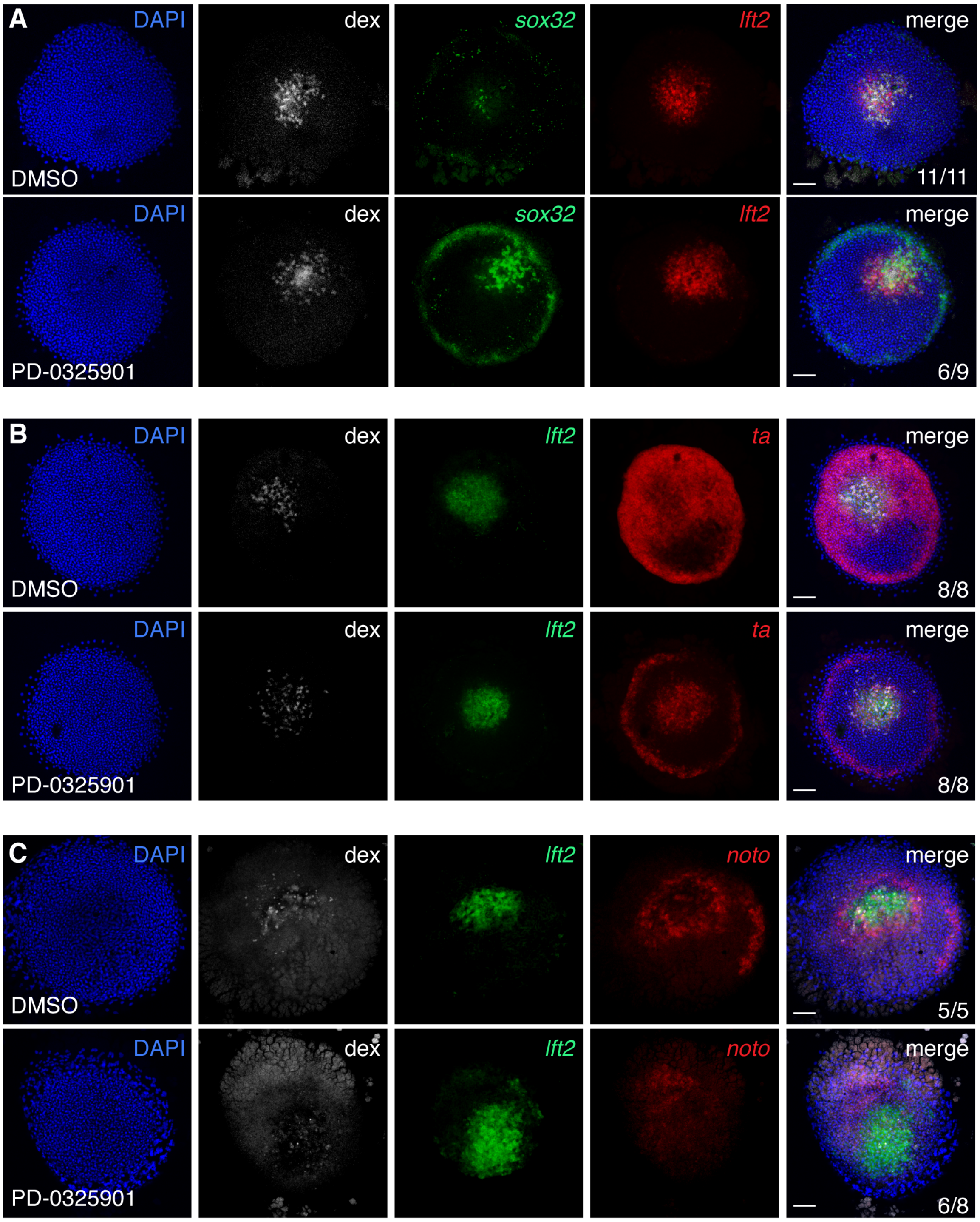


**Figure S4, related to Figure 4. Inhibition of P-Erk signaling promotes the formation of *sox32*-positive cells in Nodal-expressing clones**

(A) Flat-mounted germ ring stage embryos containing a Nodal-expressing clone treated with DMSO or MEK inhibitor PD-0325901 and stained for *sox32* and *lft2*. These are the same embryos as in Figure 4A, except that here individual channels are shown. The number of *sox32*-positive cells is strongly increased in the PD-0325901-treated embryo.

(B) As in (A), but for *lft2* and *ta*. These are the same embryos as in Figure 4D.

(C) As in (A), but for *lft2* and *noto*. These are the same embryos as in Figure 4E. In (B) and (C), incubation with PD-0325901 inhibits expression of *ta* and *noto* around the clone, confirming that this expression is due to Fgf signaling.

All scale bars are 100 μm.

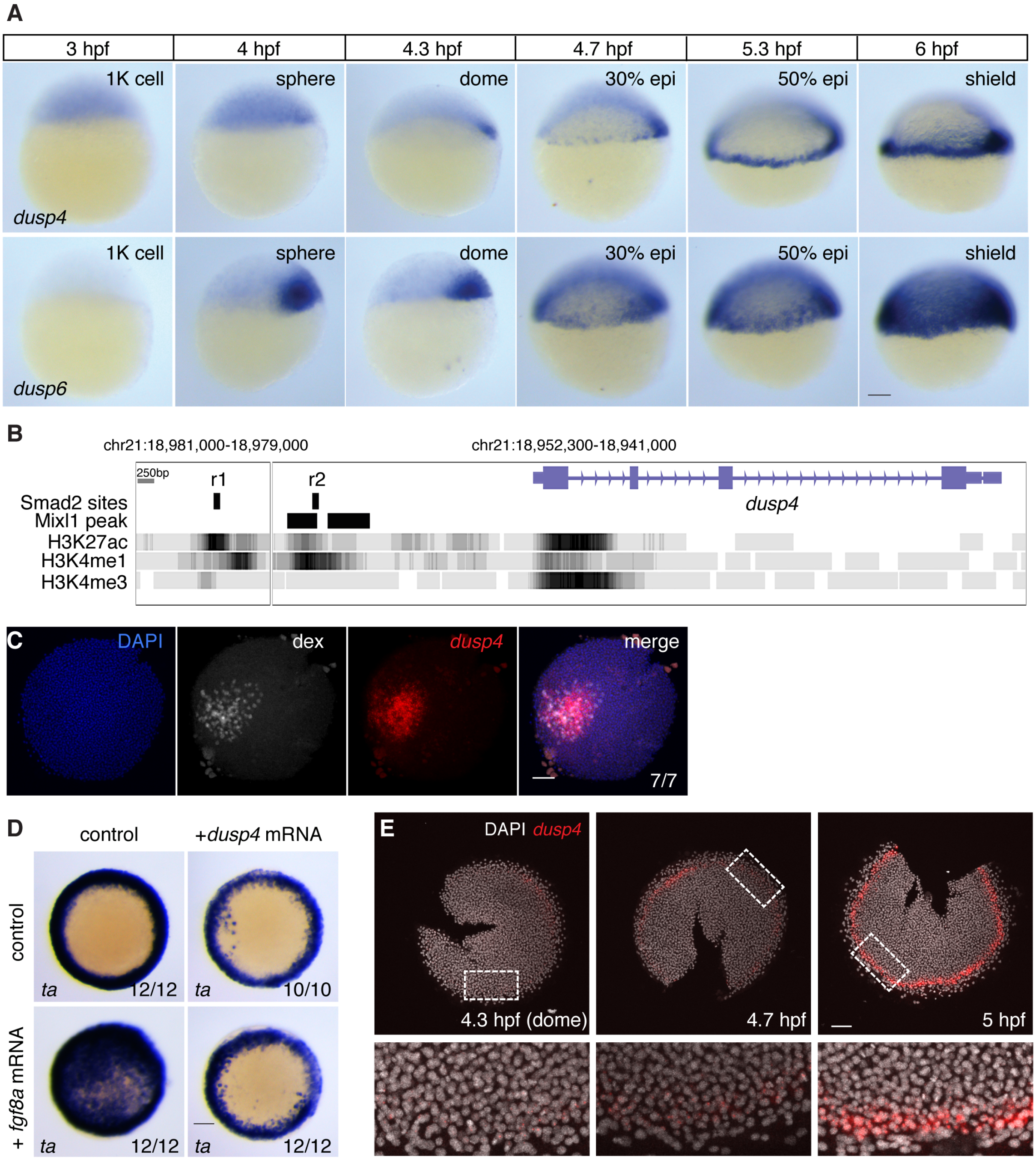


**Figure S5, related to Figure 5. Zebrafish *dusp4* is expressed in the first two cell tiers and dephosphorylates Erk**

(A) WISH for *dusp4* and *dusp6* expression in 1000-cell (1K) to shield stage embryos. Both *dusp4* and *dusp6* are initially transiently expressed in a dorsal domain, but from 30% epiboly start to be expressed in the ventral and lateral margin. *dusp4* expression remains confined up to the first cell tiers whereas *dusp6* is expressed in a much larger domain. The hours post fertilization (hpf) are indicated above.

(B) The *dusp4* locus from the UCSC genome browser (Zv9) showing the H3K27ac, H3K4me1 and H3K4me3 tracks. The positions of the putative enhancers r1 and r2 are shown, as is the position of the Mixl1 peaks taken from data reported in Nelson et al., 2017.

(C) FISH for *dusp4* mRNA surrounding Nodal-expressing clone marked with dextran fluorescein (dex). This is the same embryo shown in Figure 5G.

(D) WISH for *ta* after *fgf8a* and/or *dusp4* mRNA injection. Embryos are from the same experiment as the Western blot in Figure 5H.

(E) FISH for *dusp4* in flat-mounted embryos fixed at the times indicated to show how expression is induced over time from dome stage (4.3 hpf). Note that staining is seen in 2–3 cell tiers from the YSL at the 5 hpf. These are representative embryos from those used to calculate the traces in Figure 5I.

All scale bars are 100 µm.

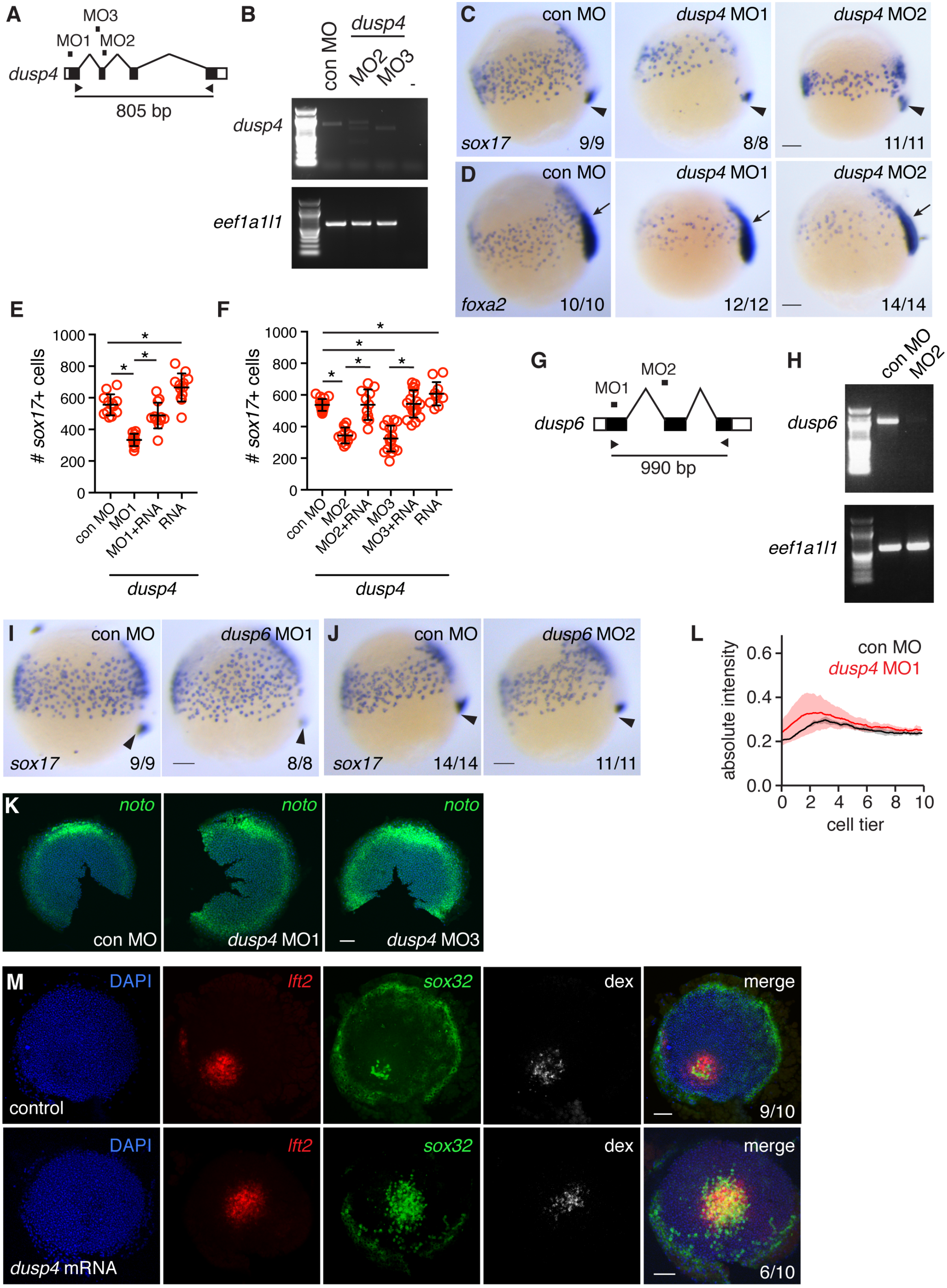


**Figure S6, related to Figure 6.** ***dusp4,*** **but not** ***dusp6*** **loss-of-function, reduces endoderm specification**

(A) Schematic representation of knockdown strategy for *dusp4* using three non-overlapping MOs (MO1–3).

(B) For MO2 and MO3, RT-PCR was performed using the indicated oligonucleotides shown in (A) to generate an 805 bp band. The housekeeping gene *eef1a1l1* was used as a control. Note that both splice blocking MOs result in loss of the 805 bp band.
(C) WISH for *sox17* on 75% epiboly embryos after injection with MO1 and MO2. Arrowhead, dorsal forerunner cells.
(D) As in (C) for *foxa2*. Note reduction of both *foxa2* and *sox17* with *dusp4* knockdown, reflecting reduction of the entire endodermal cell population (see also Figure S1B,C). Arrow, *foxa2* expression in the axial mesoderm.
(E) Quantification of endodermal cell numbers at 75% epiboly in embryos injected with control MO (con MO), *dusp4* MO1 ± 400 pg *dusp4* mRNA containing 5 silent mutations in the MO1 binding site, or with the mutated *dusp4* mRNA alone. (Means ± SD, * Mann-Whitney U test, $p < 0.05$) The MO1 phenotype is partially rescued with the mRNA.
(F) Quantification of endodermal cell numbers at 75% epiboly in embryos injected with control MO (con MO), *dusp4* MO2 or MO3 ± 400 pg *dusp4* mRNA, or with the *dusp4* mRNA alone. (Means ± SD, * Mann-Whitney U test, $p < 0.05$) The morphant phenotype is partially rescued with the mRNA.
(G) Schematic representation of knockdown strategy for *dusp6* using two non-overlapping MOs (MO1 and MO2).
(H) RT-PCR for *dusp6* after knock down with MO2.
(I) WISH for *sox17* with *dusp6* MO1 at 75% epiboly.
(J) As in (I) but using *dusp6* MO2. Neither of the *dusp6* MOs showed any effect on endodermal cell numbers. In (I) and (J), arrowhead marks dorsal forerunner cells.
(K) Flat-mounted 50% epiboly embryos stained for *noto* after knockdown of *dusp4*.
(L) Quantification of *noto* profile after injection with *dusp4* MO1 compared to control MO. For each condition, n=3, means are shown by the lines and the shading indicates the SD.
(M) Flat-mounted germ ring stage embryos containing a Nodal clone that were either control injected or injected with *dusp4* mRNA and stained for *sox32* and *lft2.* These are the same embryos as shown in Figure 6F, but here individual channels are shown.
All scale bars are 100 μm.
